# Optimization of the Antibacterial Activity of a Three-Component Essential Oil Mixture from Moroccan *Thymus satureioides*, *Lavandula angustifolia*, and *Origanum majorana* Using a Simplex–Centroid Design

**DOI:** 10.3390/ph18010057

**Published:** 2025-01-07

**Authors:** Amine Elbouzidi, Mohamed Taibi, Naoufal El Hachlafi, Mounir Haddou, Mohamed Jeddi, Abdellah Baraich, Saad Bougrine, Ramzi A. Mothana, Mohammed F. Hawwal, Waleed A. Alobaid, Abdeslam Asehraou, Bouchra El Guerrouj, Hanae Naceiri Mrabti, Francois Mesnard, Mohamed Addi

**Affiliations:** 1 Laboratoire d’Amélioration des Productions Agricoles, Biotechnologie et Environnement (LAPABE), Faculté des Sciences, Université Mohammed Premier, Oujda 60000, Morocco; mohamedtaibi9@hotmail.fr (M.T.); haddou.mounir27@gmail.com (M.H.); elguerroujb@gmail.com (B.E.G.); 2 Centre de l’Oriental des Sciences et Technologies de l’Eau et de l’Environnement (COSTEE), Université Mohammed Premier, Oujda 60000, Morocco; saad.bougrine@ump.ac.ma; 3 Faculty of Medicine and Pharmacy, Ibn Zohr University, Guelmim 81000, Morocco; naoufal.elhachlafi@usmba.ac.ma (N.E.H.); mohamed.jeddi@usmba.ac.ma (M.J.); 4 Laboratory of Bioresources, Biotechnology, Ethnopharmacology and Health, Faculty of Sciences, Mohammed First University, Boulevard Mohamed VI, B.P. 717, Oujda 60000, Morocco; abdellah.baraich@ump.ac.ma (A.B.); asehraou@yahoo.fr (A.A.); 5 Department of Pharmacognosy, College of Pharmacy, King Saud University, Riyadh 11451, Saudi Arabia; rmothana@ksu.edu.sa (R.A.M.); mhawwal@ksu.edu.sa (M.F.H.); walobaid@ksu.edu.sa (W.A.A.); 6 High Institute of Nursing Professions and Health Techniques, Casablanca 20250, Morocco; naceiri.mrabti.hanae@gmail.com; 7 Department of Pharmacology, Laboratory of Epidemiology and Research in Health Sciences, Faculty of Medicine, Pharmacy and Dentistry, Sidi Mohamed Ben Abdellah University, Fez P.O. Box 2202, Morocco; 8 BIOPI-BioEcoAgro UMRT 1158 INRAE, Université de Picardie Jules Verne, 80000 Amiens, France; francois.mesnard@u-picardie.fr

**Keywords:** *Thymus satureioides*, *Lavandula angustifolia*, *Origanum majorana*, essential oils, experimental mixture design, antibacterial activity, food preservation, biopharmaceuticals

## Abstract

Background/Objectives: The rise of antibiotic-resistant pathogens has become a global health crisis, necessitating the development of alternative antimicrobial strategies. This study aimed to optimize the antibacterial effects of essential oils (EOs) from *Thymus satureioides*, *Lavandula angustifolia*, and *Origanum majorana*, enhancing their efficacy through optimized mixtures. Methods: This study utilized a simplex–centroid design to optimize the mixture ratios of EOs for maximal antibacterial and antioxidant effectiveness. The chemical profiles of the EOs were analyzed using gas chromatography-mass spectrometry (GC-MS). The antibacterial activity was assessed against *Escherichia coli*, *Staphylococcus aureus*, and *Pseudomonas aeruginosa* using minimum inhibitory concentration (MIC) tests, while antioxidant activity was evaluated through DPPH (2,2-diphenyl-1-picrylhydrazyl), and ABTS (2,2′-azino-bis(3-ethylbenzothiazoline-6-sulfonic acid)) assays. Results: The optimized essential oil mixtures demonstrated potent antibacterial activity, with MIC values of 0.097% (*v*/*v*) for *E. coli*, 0.058% (*v*/*v*) for *S. aureus*, and 0.250% (*v*/*v*) for *P. aeruginosa*. The mixture ratios achieving these results included 76% *T. satureioides*, and 24% *O. majorana* for *E. coli*, and varying proportions for other strains. Additionally, *L. angustifolia* essential oil exhibited the strongest antioxidant activity, with IC_50_ values of 84.36 µg/mL (DPPH), and 139.61 µg/mL (ABTS), surpassing both the other EOs and standard antioxidants like BHT and ascorbic acid in the ABTS assay. Conclusions: The study successfully demonstrates that optimized mixtures of EOs can serve as effective natural antibacterial agents. The findings highlight a novel approach to enhance the applications of essential oils, suggesting their potential use in food preservation and biopharmaceutical formulations. This optimization strategy offers a promising avenue to combat antibiotic resistance and enhance food safety using natural products.

## 1. Introduction

The rise of antibiotic resistance poses a critical global health threat, with an estimated 4.95 million deaths associated with antimicrobial resistance (AMR) in 2019 alone, including 1.27 million deaths directly attributable to AMR, according to the most recent WHO Global Antimicrobial Resistance and Use Surveillance System (GLASS) report [1]. Among the most critical resistant pathogens, often referred to as “ESKAPE” pathogens, are *Enterococcus faecium*, *Staphylococcus aureus*, *Klebsiella pneumoniae*, *Acinetobacter baumannii*, *Pseudomonas aeruginosa*, and *Enterobacter* species [2]. Antibiotic-resistant bacteria pose a major health threat worldwide, leading to significant economic loss and high mortality rates, especially in developing countries, including Morocco [3,4].

Recent studies have demonstrated the significant therapeutic potential of essential oils in the fight against antibiotic-resistant bacteria [5,6]. Investigations into the synergistic effects of essential oils and conventional antibiotics have shown particularly promising results in inhibiting the growth of resistant strains [7,8]. In addition, the emergence of innovative technologies, notably nanoencapsulation, has considerably improved the physicochemical stability and antimicrobial efficacy of essential oils in the treatment of infections caused by bacterial strains [9,10].

This has led to growing interest in alternative drug sources, especially natural compounds, as numerous existing medications are derived from nature. Essential oils (EOs), which contain a blend of 20 to 100 low-molecular-weight plant secondary metabolites, are typically volatile liquids composed primarily of terpenoids, along with phenylpropanoids, benzenoids, and short-chain aliphatic hydrocarbons [11,12]. Known for their diverse bioactive composition, EOs exhibit valuable antimicrobial, antioxidant, antidiabetic, anticancer, and anti-inflammatory effects [13,14,15]. Many EOs also demonstrate significant antibacterial activity against a variety of both gram-positive and gram-negative bacteria, making them strong candidates as antimicrobial agents [16].

*Thymus satureioides* Coss., a perennial herb from the Lamiaceae family, is a well-known aromatic plant widely distributed in North Africa, particularly in Morocco, where it holds considerable ethnobotanical and medicinal value. Characterized by its small, woody structure, *T. satureioides* grows in arid and semi-arid regions, often on rocky hillsides or mountain slopes. The plant exhibits linear, lanceolate leaves, and small pink to purple flowers that bloom during spring and early summer [17]. Like many members of the *Thymus* genus, *T. satureioides* contains a variety of EOs) known for their potent bioactive properties.

In Moroccan traditional medicine, *T. satureioides* has long been used as a remedy for various ailments, particularly those associated with respiratory and digestive issues [18]. It is commonly prepared as an infusion or decoction to treat coughs, colds, bronchitis, and stomach ailments, with locals attributing these effects to its high essential oil content [19]. The plant is also employed as an antiseptic and is valued for its ability to ward off infections and promote wound healing, highlighting its cultural significance in Moroccan folk medicine.

Scientific studies have corroborated the medicinal properties attributed to *T. satureioides*, with a strong focus on its antibacterial activities. Research indicates that the essential oil of *T. satureioides* is particularly effective against various pathogenic bacteria, including *Staphylococcus aureus*, *Escherichia coli*, and *Pseudomonas aeruginosa*, which are often resistant to conventional antibiotics [20].

*Lavandula angustifolia* Mill., commonly known as lavender, belongs to the Lamiaceae family and is well-regarded for its aromatic and medicinal properties. This perennial, shrub-like plant is characterized by narrow, lanceolate leaves and tall flowering stems topped with clusters of purple-blue flowers, which bloom from late spring through summer. Lavender is native to the Mediterranean region and thrives in dry, rocky soils at moderate altitudes [21].

In Moroccan ethnobotany, *L. angustifolia* has been traditionally employed for a range of therapeutic purposes. It is widely used in Moroccan folk medicine for treating anxiety, insomnia, digestive disorders, and headaches. Lavender essential oil, known for its calming scent, is often used in aromatherapy and is applied topically to soothe skin ailments, highlighting its cultural and medicinal importance in Moroccan communities [22].

Scientific research has validated the medicinal properties attributed to *L. angustifolia*, particularly its antibacterial activities. Lavender essential oil contains various bioactive compounds, with linalool, linalyl acetate, and camphor being the most abundant constituents, responsible for the plant’s antibacterial effects [23]. Studies have demonstrated that lavender oil exhibits significant antibacterial activity against both gram-positive and gram-negative bacteria, including *S. aureus*, *E. coli*, and *P. aeruginosa* [24].

*Origanum majorana* L., commonly known as marjoram, belongs to the Lamiaceae family and is widely cultivated for its aromatic and medicinal properties. This perennial herb features soft, gray-green leaves and small white to pink flowers that bloom in clusters during the summer months. Native to the Mediterranean region, *O. majorana* thrives in sunny, well-drained soils and is often grown in dry, rocky landscapes [25]. In Moroccan ethnobotany, *O. majorana* is valued for its therapeutic applications in traditional medicine. Known locally as “mardakouch”, it is used to relieve respiratory ailments, digestive disorders, and muscle pain and is believed to have calming effects when consumed as an infusion. This cultural significance is rooted in its broad application in Moroccan households for both culinary and medicinal purposes, where it is commonly prepared as a tea or used in decoctions [26].

Studies have shown that marjoram essential oil exhibits potent antibacterial activity against both gram-positive and gram-negative bacteria, including *E. coli*, *Salmonella typhi*, and *S. aureus* [27]. The antibacterial mechanisms of *O. majorana* essential oil are thought to involve the disruption of bacterial cell walls and membranes, ultimately leading to cell lysis [28].

Beyond their antimicrobial properties, EOs have gained considerable attention for their antioxidant potential. This is particularly relevant given that oxidative stress, characterized by an imbalance between reactive oxygen species (ROS) production and cellular antioxidant defenses, plays a crucial role in the pathogenesis of numerous diseases, including cardiovascular disorders, neurodegenerative conditions, and cancer [29]. The accumulation of ROS can cause significant damage to cellular components, including lipids, proteins, and DNA, leading to cellular dysfunction and death [30]. Natural antioxidants, particularly those found in EOs, have emerged as promising therapeutic alternatives due to their ability to neutralize free radicals and protect against oxidative damage [31,32].

Research has shown that combining EOs can yield synergistic effects, enhancing efficacy against pathogens more effectively than single applications. However, most studies have focused on simple binary or ternary EO mixtures without optimization. The simplex–centroid-mixture design allows researchers to model and predict the behavior of mixture components and their interactions using a minimal number of experimental runs while maintaining high accuracy [33,34]. Unlike traditional one-factor-at-a-time approaches, simplex–centroid designs can efficiently explore the entire mixture space and identify optimal blend proportions, making them particularly valuable for studying EO combinations [35].

This study employs a simplex–centroid-mixture design to optimize the antibacterial effects of EOs from *T. satureioides*, *L. angustifolia*, and *O. majorana*, three plants with established ethnobotanical significance in Morocco. The antibacterial activities of both individual EOs and their mixtures are assessed through this design approach, while their antioxidant properties are evaluated individually, with the goal of comprehensively characterizing their therapeutic potential.

## 2. Results and Discussion

### 2.1. Chemical Profile of the Three EOs

Table 1 provides a comprehensive overview of the chemical profiles, molecular formulas, percentages, and extraction yields of EOs derived from *T. satureioides*, *L. angustifolia*, and *O. majorana*. Detailed analytical data, including the Total Ion Chromatogram (TIC) chromatograms, compositional breakdowns, and retention times, are presented in the Supplementary Materials (Figures S1–S3 and Tables S1–S3). The extraction yields of the EOs were determined to be 2.34% (*v*/*w*) for *T. satureioides*, 1.68% (*v*/*w*) for *L. angustifolia*, and 3.45% (*v*/*w*) for *O. majorana*. Each essential oil displayed a distinct phytochemical composition, with *T. satureioides* containing 29 phytoconstituents, *L. angustifolia* comprising 21, and *O. majorana* including 23. Collectively, these identified components accounted for 100% of the total chemical composition of the respective EOs.

In *T. satureioides* EO (TSEO), sesquiterpenes are dominant, particularly β-himachalene (42.16%) and α-himachalene (20.04%), which contribute to the oil’s therapeutic profile. Both compounds are widely recognized for their anti-inflammatory and antimicrobial activities, positioning TSEO as a potentially valuable medicinal agent. Caryophyllene, another sesquiterpene, is present at 10.80%, adding to the oil’s bioactivity spectrum with its documented anti-inflammatory properties, as supported by studies showing its efficacy in pain and inflammation management [36]. Minor compounds like δ-cadinene (1.45%) and tumerone (1.19%) also contribute to the complexity of TSEO’s bioactivity. This profile aligns with studies on *Thymus* species in North Africa, where sesquiterpenes are often predominant in *Thymus* EOs, particularly under arid conditions, suggesting ecological factors may influence the profile [37].

The EO of *L. angustifolia* (LAEO) is primarily composed of oxygenated monoterpenes, with linalool (19.12%) and 1,8-cineole (15.42%) as major components. Linalool, a compound noted for its calming and antimicrobial effects, significantly contributes to LAEO’s therapeutic potential and is frequently cited in aromatherapy studies [38]. Similarly, 1,8-cineole has been widely reported in *L. angustifolia* oils and is valued for its antimicrobial and respiratory benefits, which makes LAEO a versatile EO for health and wellness applications. In comparison, a study by Guitton et al. (2018) found 1,8-cineole at comparable levels in *Lavandula* species, highlighting its consistent occurrence across this genus [39]. camphor (10.02%) and borneol (5.66%) are also prevalent, both of which are known for antimicrobial, analgesic and stimulant properties [40,41,42].

*O. majorana* EO (OMEO) is particularly rich in camphor (39.58%) and 3-thujanone (23.03%), indicating its potential use in antimicrobial and anti-inflammatory applications. Camphor, a terpene with antiseptic and mild anesthetic effects, has shown efficacy in various medicinal applications, as noted in research on *Origanum* species across the Mediterranean [43]. The presence of thujone (5.69%) and epiglobulol (5.50%) further enhances OMEO’s unique aroma and potential therapeutic benefits. These compounds provide a balanced profile of oxygenated monoterpenes and monoterpenes, which contributes to OMEO’s popularity in both medicinal and culinary contexts. This profile aligns with findings from Aligiannis et al. (2001) [44], which similarly observed high camphor content in Mediterranean *Origanum* species.

Comparing these oils, some compounds such as β-myrcene, linalool, and γ-terpinene are found across all three EOs, albeit in varying concentrations. For instance, linalool appears most abundantly in LAEO (19.12%), while it is present minimally in TSEO (0.72%), and OMEO (0.80%). Camphor, present in both LAEO and OMEO, contributes to their shared therapeutic versatility, with studies showing its benefits in respiratory and pain-relieving applications [45]. In contrast, β-himachalene is highly specific to TSEO, emphasizing its distinctive sesquiterpene profile.

**Figure 1:**
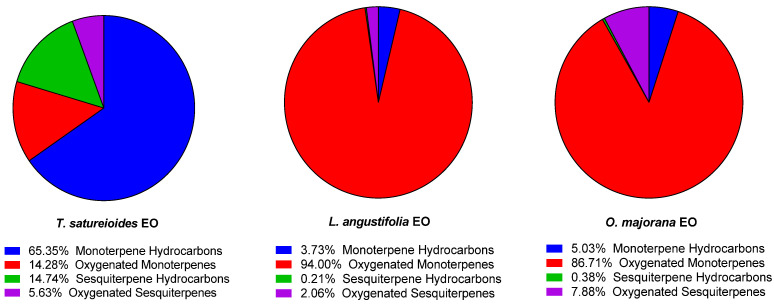
Chemical diversity of sub-classes of terpenes found in the studied essential oils from *T. satureioides*, *L. angustifolia*, and *O. majorana*.

In terms of structural group composition, TSEO is notably rich in monoterpenes hydrocarbons (65.35%), and sesquiterpene hydrocarbons (14.74%), while LAEO and OMEO are mainly dominated by oxygenated monoterpenes (94.00%, and 86.71%, respectively) (Figure 1). These results are in accordance with previously published studies on these plants [46,47,48]. This distribution reflects their differing ecological adaptations and potential applications. Sesquiterpenes, being more stable and less volatile, are particularly suited for applications requiring prolonged activity, such as their incorporation in smart packaging treatments [49].

Overall, these investigations highlight the critical role of ecological, climatic, and nutritional factors in shaping both the quantitative and qualitative composition of EOs in plants. They provide compelling evidence that the chemical profiles of EOs are influenced by external and internal factors, including climatic conditions, seasonal fluctuations, soil properties, and intrinsic metabolic pathways. These findings emphasize the complex interplay between environmental conditions and plant physiology in determining the unique chemical characteristics of EOs [50].

### 2.2. Antioxidant Activity of Individual EOs

**Figure 2:**
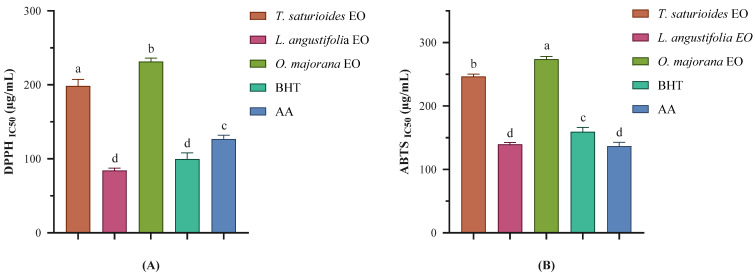
The antioxidant activity of the EOs was assessed using the DPPH assay (**A**) and the ABTS test (**B**), with butylated hydroxytoluene (BHT) and ascorbic acid (AA) serving as reference standards. Results are expressed as the mean ± standard deviation (SD) from three independent experiments. Statistically significant differences between groups are denoted by different letters, with significance established at *p* < 0.05.

The antioxidant potential of the EOs derived from *T. satureioides*, *L. angustifolia*, and *O. majorana* was assessed using two well-established assays: the DPPH and ABTS radical scavenging methods (Figure 2). These tests are complementary, as they evaluate antioxidant activity through distinct mechanisms and in varying reaction environments [51,52]. All three EOs demonstrated greater antioxidant efficacy in the ABTS assay compared to the DPPH assay. This variation can be attributed to the differences in assay characteristics: ABTS is suitable for both hydrophilic and lipophilic antioxidant systems, whereas DPPH is primarily effective for hydrophobic systems [53,54].

To classify the antioxidant activity of essential oils, IC_50_ values obtained from the DPPH assay were used as benchmarks. Essential oils with IC_50_ values between 200 and 300 µg/mL were categorized as exhibiting moderate antioxidant activity. This classification is supported by previously published studies, including El Hachlafi et al. (2023), which established similar thresholds for evaluating antioxidant potency in essential oils [55].

*T. satureioides* EO displayed moderate antioxidant activity, with IC_50_ values of 284.67 ± 2.58 µg/mL in the DPPH assay and 239.54 ± 3.29 µg/mL in the ABTS assay. This EO’s chemical composition includes high concentrations of β-himachalene (42.16%), α-himachalene (20.04%), caryophyllene (10.80%), and thymol (9.71%). Both β-himachalene and α-himachalene are sesquiterpenes, and while sesquiterpenes are generally known for their anti-inflammatory and antimicrobial properties, their antioxidant activity can vary. Research on the antioxidant activity of himachalene derivatives is limited; however, sesquiterpenes like these may offer moderate antioxidant effects due to their ability to interact with lipid radicals and slow down oxidative processes [56]. Caryophyllene is a bicyclic sesquiterpene and is known for its significant antioxidant activity. Studies have shown that caryophyllene can scavenge free radicals effectively, likely due to its unique structure, which allows it to donate electrons and stabilize radical species [57]. Thymol, a monoterpenoid phenol, is well-known for its potent antioxidant properties, attributed to its ability to donate hydrogen atoms to free radicals and stabilize them. Thymol’s hydroxyl (-OH) group enables it to act as a strong radical scavenger, making it one of the more effective compounds for preventing oxidative damage [43,58].

Among the tested EOs, *L. angustifolia* displayed the most potent antioxidant activity, with IC_50_ values of 84.36 ± 2.99 µg/mL in the DPPH assay and 139.61 ± 2.82 µg/mL in the ABTS assay. Notably, in the DPPH assay, the antioxidant performance of LAEO (IC_50_ = 84.36 ± 2.99 µg/mL) exceeded that of synthetic antioxidants, such as BHT (IC_50_ = 99.72 ± 8.42 µg/mL) and ascorbic acid (AA) (IC_50_ = 126.78 ± 5.33 µg/mL). The EO also showed good activity in the ABTS assay (IC_50_ = 139.61 ± 2.82 µg/mL). This exceptional activity is consistent with findings from earlier studies, including those by Nikšić et al., who reported a high antioxidant capacity for *L. angustifolia* EO, with an IC_50_ value of 0.421 ± 0.03 mg/mL [59].

The superior antioxidant activity of *L. angustifolia* EO can be attributed to its unique chemical composition. The EO contains high concentrations of linalool (19.12%), 1,8-cineole (15.42%), thymol (12.57%), and camphor (10.02%), each of which is known for contributing to antioxidant effects. Linalool, a major component of *L. angustifolia* EO, is a monoterpene alcohol with established antioxidant properties. Linalool’s hydroxyl (-OH) group can donate hydrogen atoms to neutralize free radicals, thus stabilizing them and reducing oxidative stress. This property has been well-documented in the literature, where linalool is often highlighted for its ability to scavenge radicals, particularly in EOs from lavender species [23]. Camphor, another component of *L. angustifolia* EO, is a bicyclic monoterpene ketone with moderate antioxidant properties. While camphor demonstrates lower radical scavenging capacity compared to linalool and thymol, it may contribute to antioxidant activity through its ability to modulate lipid peroxidation mechanisms [60].

*O. majorana* EO demonstrated strong antioxidant potential, with IC_50_ values of 231.57 ± 4.57 µg/mL in the DPPH assay and 273.91 ± 4.36 µg/mL in the ABTS assay. This result is consistent with findings from Milos et al. (2000), who reported significant free radical scavenging activity in *O. majorana* EO in both DPPH and ABTS assays, attributing its antioxidant properties to a rich chemical profile that includes high concentrations of oxygenated monoterpenes, such as terpinen-4-ol and γ-terpinene [61].

Camphor, the most abundant component at 39.58%, has been reported to exhibit robust free radical scavenging activity (in DPPH test, an IC_50_ = 77.00 ± 8.30 µg/mL; an IC_50_ of 96.32 4.15 µg/mL in ABTS test, and an IC_50_ of 101.12 ± 4.03 µg/mL in FRAP test), significantly contributing to the EO’s overall antioxidant performance [62]. This EO also contains 3-thujanone (23.03%), 1,8-cineol (7.42%), and thujone (5.69%), both of which are recognized for their antioxidative effects [63]. The synergy among these active compounds likely enhances the antioxidant efficacy of *O. majorana* EO, positioning it as a valuable natural source of antioxidants for potential applications in food preservation and healthcare.

Terpenoids, including compounds found in these EOs, possess significant antioxidant potential, primarily through their ability to neutralize free radicals. Oxidative stress arises when there’s an imbalance between free radicals, or reactive oxygen species (ROS), and the body’s antioxidant defenses, leading to cellular damage. Terpenoids play a vital role in combating oxidative stress, making them valuable in both traditional and modern therapeutic applications [64].

### 2.3. Single Antibacterial Activity

Table 2 highlights the antibacterial activity of three EOs (TSEO, LAEO, OMEO) against *E. coli*, *S. aureus*, and *P. aeruginosa*, with comparisons to the antibiotics Kanamycin and Chloramphenicol. The inhibition zone diameters (IZ) and minimum inhibitory concentrations (MIC) reveal the varying effectiveness of these EOs in inhibiting bacterial growth.

Firstly, examining the inhibition zone diameters, TSEO stands out as the most potent, especially against *S. aureus* (35.05 ± 1.95 mm), followed by *E. coli* (26.80 ± 0.30 mm), and *P. aeruginosa* (30.25 ± 0.75 mm). This aligns with the existing literature suggesting that thyme oil, rich in monoterpene hydrocarbons, is particularly effective against gram-positive bacteria such as *S. aureus* due to its ability to disrupt bacterial cell membranes [28]. In comparison, LAEO shows moderate IZ values, being most effective against *S. aureus* (19.75 ± 1.75 mm), and less so against *E. coli* (12.70 ± 1.54 mm) and *P. aeruginosa* (15.50 ± 3.00 mm). This effect can be attributed to lavender oil’s components, such as linalool and linalyl acetate, which have known antimicrobial properties, especially against gram-positive bacteria [65]. Meanwhile, OMEO demonstrates limited activity with minimal IZ values across all bacteria, particularly against *S. aureus,* with an IZ value of 1.64 ± 0.50 mm. Though oregano oil usually contains oxygenated monoterpenes, with 1,8-cineol, thymol, and camphor, and is effective against bacterial pathogens, as reported by Si et al. (2006) [66].

The minimum inhibitory concentration (MIC) values further reinforce these findings. TSEO exhibits the lowest MIC values (0.25–0.5% *v*/*v*) across all bacterial strains, highlighting its strong antibacterial effect. Lower MIC values for TSEO against *S. aureus*, and *E. coli* suggest that even at low concentrations, thyme oil can effectively inhibit bacterial growth, consistent with its high IZ values. LAEO, on the other hand, requires higher concentrations to achieve inhibition (MIC of 1–2% *v*/*v*), indicating moderate antibacterial potency. Its MIC values are similar to those of OMEO, suggesting that while LAEO can be effective, it requires higher concentrations than TSEO to achieve similar effects. OMEO, with MIC values of 2% *v*/*v* across all bacteria, demonstrates limited antibacterial efficacy, aligning with its small IZ values and indicating that it might not be as potent or might contain fewer active components.

Comparing these results with the antibiotics kanamycin and chloramphenicol, it is clear that the synthetic antimicrobials exhibit stronger antibacterial activity. Chloramphenicol, in particular, shows the lowest MIC values (0.062–0.25% *v*/*v*) and consistently large IZs across all bacterial strains, underscoring its superior potency. While TSEO’s activity is notable and, in some cases, comparable to kanamycin, the antibiotics’ overall effectiveness is much higher. Nevertheless, with rising antibiotic resistance, EOs like TSEO present a promising area for developing complementary or alternative antimicrobial agents [67].

The antibacterial activity of these EOs can be attributed to their specific chemical compositions and associated mechanisms of action. These bioactive compounds target bacterial cells through diverse pathways, including membrane disruption, interference with enzymatic activities, and induction of oxidative stress, leading to inhibiting growth and cell death. For instance, β-himachalene is known for its high affinity to the main bacterial protease, suggesting a potential role in inhibiting bacterial protein functions [68]. Caryophyllene is known to alter membrane permeability and integrity, causing extensive damage to bacterial cell membranes. This leads to leakage of intracellular content, which disrupts cellular homeostasis and results in cell death [69]. Similarly, carvacrol and thymol are potent antibacterial agents against both gram-positive and gram-negative bacteria, disrupting bacterial membranes and inhibiting membrane ATPases [70]. Additionally, Linalool has been shown to disrupt the bacterial membrane by inducing oxidative stress, leading to intracellular leakage and cell death [71]. Furthermore, Oxygenated monoterpenes such as cineol contribute to antibacterial efficacy by damaging bacterial membranes and inducing reactive oxygen species (ROS)-mediated oxidative stress [72].

These findings support the potential of EOs, particularly TSEO, as natural antimicrobials, especially in applications where antibiotic resistance is a concern. However, further research is necessary to understand the mechanisms of action, synergistic effects with antibiotics, and in vivo efficacy of these oils. Additionally, the variability in efficacy among different EOs suggests a need for standardized extraction and formulation processes to ensure consistent antibacterial performance [73].

### 2.4. Simplex Centroid Design

Table 3 provides a detailed examination of the antibacterial effectiveness of various combinations of EOs from *T. satureioides*, *L. angustifolia*, and *O. majorana* against three bacterial strains: *E. coli*, *S. aureus*, and *P. aeruginosa*. This study employs a simplex–centroid design to evaluate the synergistic effects of these oils, with effectiveness quantified by the minimum inhibitory concentration (MIC).

The MIC values across the table suggest varying degrees of antibacterial activity. For *E. coli*, the MIC ranges from 0.25 to 0.5, indicating that certain oil combinations can effectively inhibit this gram-negative bacterium. Literature supports this finding, particularly highlighting the efficacy of *O. majorana* EO due to its compounds which are known to disrupt bacterial cells [74].

Against *S. aureus*, the MIC values span from 0.25 to 1.0. This variation could be attributed to the different active components in the oils and the unique cell wall properties of this gram-positive bacterium. *L. angustifolia* EO is notably effective, potentially due to its linalool content that can penetrate and disrupt bacterial membranes [23].

The challenge of inhibiting *P. aeruginosa* is evident with MIC values ranging from 0.375 to 1.0, reflecting this pathogen’s notorious resistance to many antibiotics. The contribution of *T. satureioides* EO, rich in monoterpene hydrocarbons, including thymol and carvacrol, is significant here as these components are known for their ability to compromise the integrity of resistant bacterial membranes [75].

The study also explores balanced and specialized mixtures of these oils. For instance, mixtures with equal proportions of all three oils show broad-spectrum effectiveness, suggesting potential for general antibacterial applications. Conversely, mixtures tailored with higher proportions of specific oils, such as *L. angustifolia* for targeting *S. aureus*, indicate the possibility of optimizing these blends for particular therapeutic uses against resistant strains.

### 2.5. Variance Analysis of the Fitted Models

Table 4 presents the variance analysis used to evaluate the regression models for antimicrobial activity of essential oil mixtures against *E. coli*, *S. aureus*, and *P. aeruginosa*. The analysis examined degrees of freedom (DF), sum of squares (SS), mean squares (MS), F-statistics, and *p*-values for each bacterial species.

**Figure 3:**
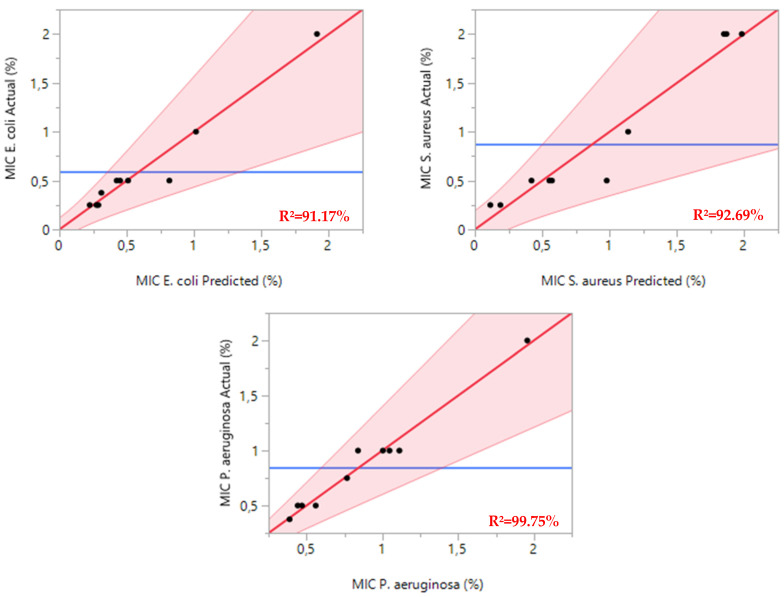
MIC responses against *E. coli*, *S. aureus*, and *P. aeruginosa* are represented by curves illustrating the relationship between the experimental values and the expected values, depicted by red lines. Meanwhile, the blue lines indicate the actual mean values for the two responses under investigation.

For *E. coli*, the regression model showed strong statistical significance with an F-statistic of 16.0996 and a *p*-value of 0.0039. The model achieved an R^2^ of 0.9117, indicating that it effectively explains over 91% of the variance in minimum inhibitory concentrations (MICs) (Figure 3.). This high explanatory power confirms the model’s reliability in predicting the antibacterial effects of essential oil combinations against *E. coli*.

The model for *S. aureus* demonstrated similar robustness, with an F-statistic of 12.5836 and a *p*-value of 0.0069. The R^2^ value of 0.9269 suggests excellent predictive accuracy, validating the model’s ability to characterize how essential oil mixtures affect *S. aureus* growth inhibition.

The most striking results emerged from the *P. aeruginosa* analysis, which yielded an F-statistic of 32.0945 and a *p*-value of 0.0008. The exceptionally high R^2^ of 0.9975 demonstrates near-perfect alignment between predicted and observed MIC values, confirming the model’s outstanding accuracy in predicting the antimicrobial efficacy of essential oil combinations against this notably antibiotic-resistant pathogen.

### 2.6. Components Effects and Adjusted Models

The computed regression coefficients for the special model are shown in Table 5. The associations between all tested parameters and the obtained MIC responses for *E. coli*, *S. aureus*, and *P. aeruginosa* were found using regression models with significant coefficients (*p*-values < 0.05).

The MIC response against *E. coli* reveals that the binary interaction terms α13 and α23, along with the coefficients representing the effects of individual components (α_2_ and α_3_), are statistically significant. However, the ternary interaction term (α_123_), the binary interaction term α_12_, and the linear term (α_1_) do not exhibit any significant influence on the E. coli response (*p* > 0.05). Consequently, these non-significant coefficients were excluded from the proposed models, and the resulting mathematical model, as expressed in Equation (1), describes the response as a function of the significant components.

(1) $$Y_{\;MIC-E.\;\;coli}={1.013X}_{2}+{1.91X}_{3}-3.12X_{1}X_{3}-4.15X_{2}X_{3}+ɛ$$

Concerning the MIC*_S. aureus_* response, the significant terms were α_1_, α_13_, and α_23_. These results confirm that the binary effect *T. satureioides*, and *L. angustifolia* EOs have a major influence on the antibacterial activity against *S. aureus*. Equation (2) thus expresses the accepted mathematical model:

(2) $$Y_{\;MIC-S.\;\;aureus}={1.86X}_{2}+{1.98X}_{3}-4.12X_{1}X_{2}+ɛ$$

For the MIC*_P. aeruginosa_* response, the significant terms were α_1_, α_2_, α_3_, α_13_, and α_23_. These results showcase that linear and binary terms (except for α_12_) have a major influence on the antibacterial activity against *P. aeruginosa*. Equation (3) thus expresses the accepted mathematical model:

(3) $$Y_{\;MIC-P.\;\;aeruginosa}={0.38X}_{1}+{1.04X}_{2}+{1.95X}_{3}-2.81X_{1}X_{3}-1.99X_{2}X_{3}+ɛ$$

### 2.7. Desirability Functions and Optimization of the Mixture

The optimization process, guided by experimental design methodology, aims to identify the ideal ratios of the studied components to maximize the response values. While the optimal results predicted by statistically validated mathematical models may not always precisely match those observed in the 12 experimental trials, they reliably estimate response values within the experimental range. To determine the most favorable outcomes, the optimization process begins by evaluating the highest recorded values. Accordingly, the best MIC results were 0.25%, 0.25%, and 0.375% *v*/*v* for *E. coli*, *S. aureus*, and *P. aeruginosa*, respectively. Any configurations capable of producing responses at or above these levels were considered acceptable.

### 2.8. Formulation Profile

**Figure 4:**
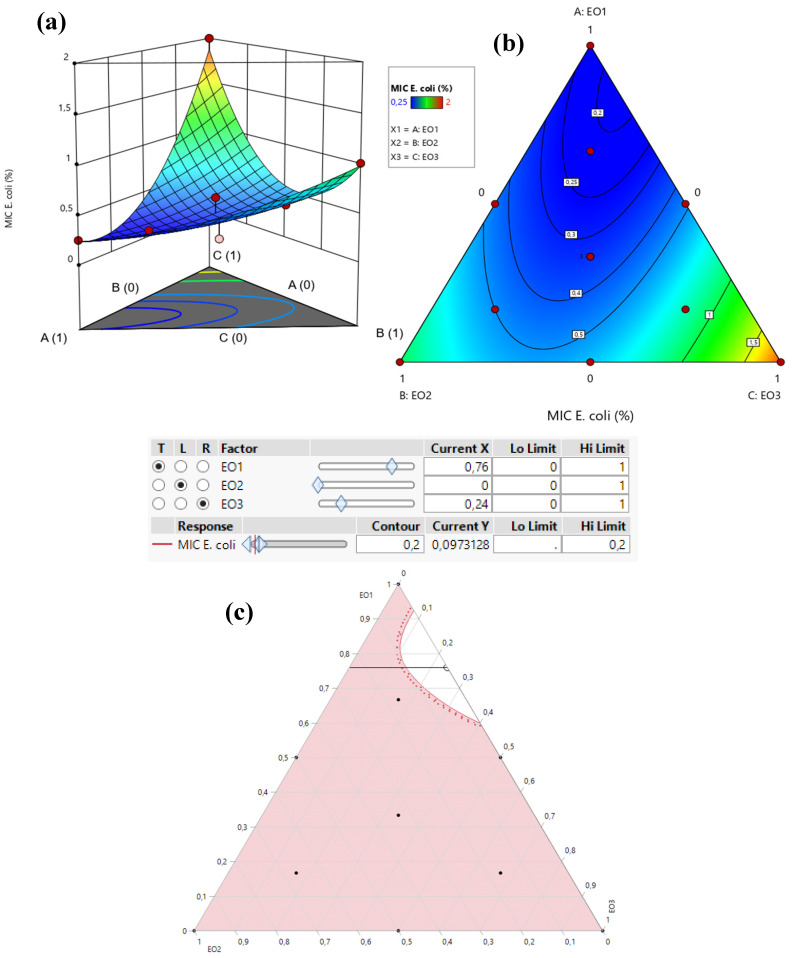
The optimal MIC value for *E. coli* was determined through an in-depth analysis of 2D and 3D mixture plots, focusing on the identified compromise zone. Panels (**a**,**b**) display 3D mixture plots that highlight the desired compromise region, located within the binary mixing zone between *T. satureioides* and *O. majorana*. This zone represents the optimal conditions for achieving maximum antibacterial activity. Panel (**c**) further illustrates this relationship through a 2D mixture plot, pinpointing the specific proportions of the EOs required to reach the desired MIC value of 0.097% against the *E. coli* strain. The optimal composition was achieved with a mixture consisting of 76% *T. satureioides*, and 24% *O. majorana* EOs. EO1: *T. satureioides* EO; EO2: *L. angustifolia* EO; EO3: *O. majorana* EO.

**Figure 5:**
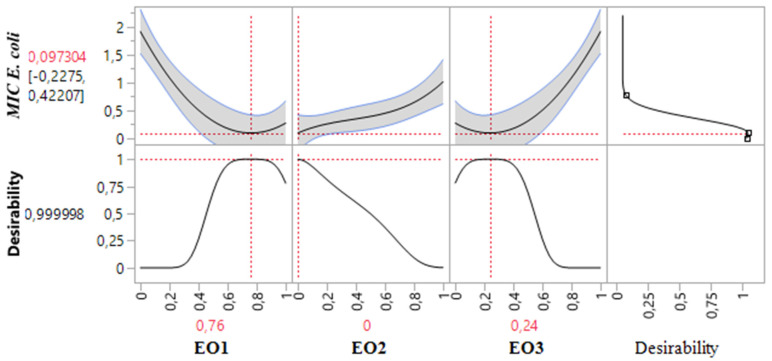
Desirability profile illustrating the precise proportions, leading to the optimum value for MIC*_E.coli_.* EO1: *T. satureioides* EO; EO2: *L. angustifolia* EO; EO3: *O. majorana* EO.

The contour plot and the 3D surface graph, depicted as 2D and 3D mixture plots in Figure 4 and Figure 5, illustrate the optimal combination of the three EOs—*T. satureioides*, *L. angustifolia*, and *O. majorana*—for maximizing antibacterial activity, measured as MIC responses against *E. coli*, *S. aureus*, and *P. aeruginosa*. These visual tools provide a clear representation of the relationship between the MIC responses and the concentrations of each essential oil. Generated using Design-Expert software v12.0, the plots employ iso-response curves, which are particularly effective for identifying the precise conditions necessary to achieve the most favorable response values. In the visualizations, the color gradients represent varying levels of antibacterial activity. The blue areas correspond to the lowest MIC values, indicating the highest antibacterial efficacy. In contrast, the regions shaded from yellow to dark red indicate progressively higher MIC values, reflecting reduced antibacterial effectiveness.

#### 2.8.1. Optimization of MIC*_E. coli_* Response

The 2D and 3D mixture plots (Figure 4) reveal that the dark blue region corresponds to the optimal compromise for achieving the lowest MIC value for *E. coli*, determined to be 0.20% *v*/*v*. This optimal result is achieved using a binary combination of *T. satureioides* and *O. majorana* EOs. The efficacy of this specific mixture is further validated by the desirability test (Figure 5), which confirms that a MIC value of 0.20% *v*/*v*, with an impressive desirability score of 99.99%, can be attained when the EOs are combined in the following proportions: 76% *T. satureioides* and 24% *O. majorana*.

These findings highlight the efficiency of this binary mixture in delivering significant antibacterial activity against *E. coli*, emphasizing the importance of precise component ratios in optimizing response values. The integration of contour and desirability analysis provides robust evidence supporting the optimal formulation, which holds potential for targeted antibacterial applications.

#### 2.8.2. Optimization of MIC*_S. aureus_* Response

**Figure 6:**
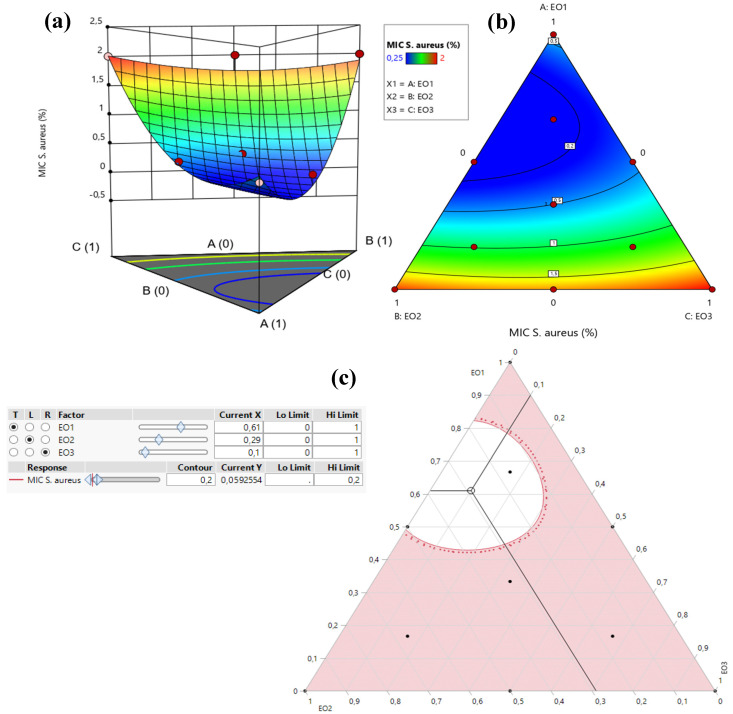
The optimal MIC value for *S. aureus* was determined through an analysis of 2D and 3D mixture plots focused on the identified compromise region. Panels (**a**,**b**) illustrate 3D mixture plots, highlighting the desired compromise zone within the ternary mixing area of *T. satureioides*, *L. angustifolia*, and *O. majorana*. Panel (**c**) presents a 2D mixture plot, which identifies the optimal compromise region leading to the desired MIC value of 0.058% against *S. aureus*. This result was achieved by using a ternary mixture composed of 61% *T. satureioides*, 29% *L. angustifolia*, and 10% *O. majorana* essential oils. EO1: *T. satureioides* EO; EO2: *L. angustifolia* EO; EO3: *O. majorana* EO.

The 2D and 3D mixture plots (Figure 6) illustrate the dark blue region as the optimal compromise zone for achieving the lowest MIC value for *S. aureus*. Experimental MIC values for *S. aureus* ranged from 0.25% to 2% *v*/*v*, as shown in Table 3. Analysis of the contour and surface plots (Figure 6) indicates that an optimized MIC value of 0.058% *v*/*v* can be achieved through a ternary combination of EOs comprising 61% TSEO, 29% LAEO, and 10% OMEO.

**Figure 7:**
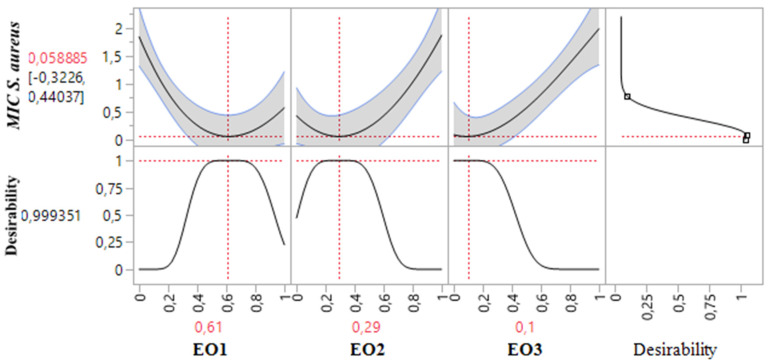
Desirability profile illustrating the precise proportions leading to the optimum value for MIC*_S.aureus_*. EO1: *T. satureioides* EO; EO2: *L. angustifolia* EO; EO3: *O. majorana* EO.

This optimization is further supported by the desirability function (Figure 7), which demonstrates a 99.93% probability of attaining the ideal MIC value (0.058% *v*/*v*) with this specific combination. These findings highlight the effectiveness of ternary mixtures in achieving superior antibacterial activity against *S. aureus* and emphasize the value of statistical and graphical tools in identifying optimal component ratios for enhanced response outcomes.

#### 2.8.3. Optimization of MIC*_P. aeruginosa_* Response

**Figure 8:**
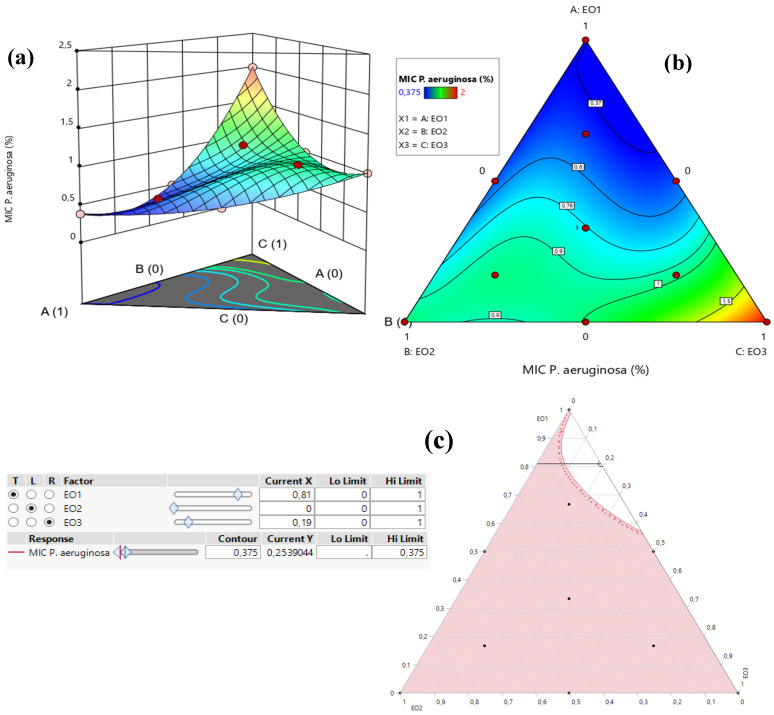
The optimal MIC value for *P. aeruginosa* was determined through an analysis of 2D and 3D mixture plots focused on the identified compromise region. Panels (**a**,**b**) depict 3D mixture plots, highlighting the desired compromise zone within the binary mixing area of *T. satureioides* and *O. majorana*. Panel (**c**) presents a 2D mixture plot that identifies the optimal compromise region, achieving the target MIC value of 0.25% against *P. aeruginosa*. This result was attained with a binary mixture comprising 81% *T. satureioides* and 19% *O. majorana* essential oils. EO1: *T. satureioides* EO; EO2: *L. angustifolia* EO; EO3: *O. majorana* EO.

Table 3 demonstrates that the MIC response for *P. aeruginosa* varied between 0.375% and 2%. An analysis of the contour and surface plots (Figure 8) indicates that achieving a significantly low MIC value of 0.02% necessitates a binary mixture of TSEO and OMEO. This highlights the efficacy of these two EOs in combination for strong antibacterial action against *P. aeruginosa*.

**Figure 9:**
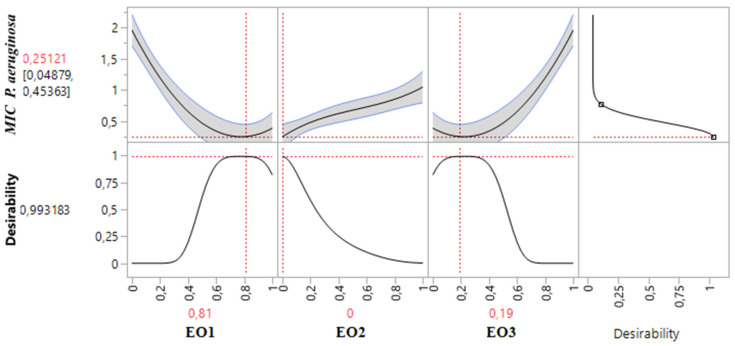
Desirability profile illustrating the precise proportions leading to the optimum value for MIC*_P. aeruginosa_*. EO1: *T. satureioides* EO; EO2: *L. angustifolia* EO; EO3: *O. majorana* EO.

Additionally, the desirability function analysis (Figure 9) provides further insights, suggesting a 99.31% probability of achieving an optimal MIC value of 0.25% by utilizing a binary mixture primarily composed of 81% TSEO and 19% OMEO. These results underscore the importance of precise ratio optimization in enhancing the antibacterial potency of essential oil mixtures, providing a practical framework for achieving superior outcomes against *P. aeruginosa*.

### 2.9. Simultaneous Optimization of All Responses

The results of simultaneous optimization are particularly evident in the contour plots illustrating the MIC responses for *S. aureus*, *P. aeruginosa*, and *E. coli*, influenced by the combinations of TSEO, LAEO, and OMEO. Notably, the optimal formulations derived from this study demonstrated significantly enhanced antibacterial activity compared to the individual pure EOs, underscoring the efficacy of these optimized mixtures.

**Figure 10:**
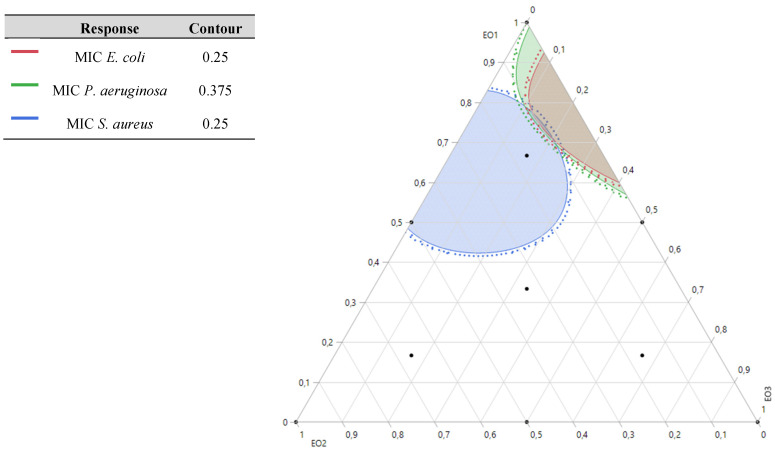
2D mixture contour plot of the optimal combination region between EOs, resulting in the best value of MIC for *E. coli*, *S. aureus*, and *P. aeruginosa*.

**Figure 11:**
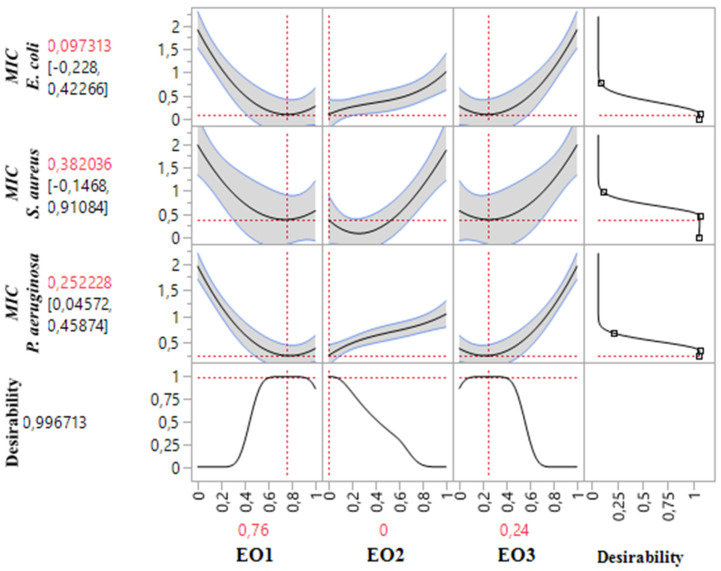
EDesirability profiles of the simultaneous optimization of all responses yielding an optimal mixture of 76% of EO1 (*T. satureioides*), 0% EO2 (*L. angustifolia*), and 24% of EO3 (*O. majorana*).

These findings validate the performance of the formulated combinations. For *S. aureus*, the desired compromise region to achieve the target MIC requires a ternary mixture predominantly composed of TSEO, LAEO, and OMEO. In contrast, for *E. coli* and *P. aeruginosa*, the optimal antibacterial activity is achieved with a binary mixture primarily comprising TSEO and OMEO. These results, depicted in Figure 10 and Figure 11, highlight the critical role of precise component ratios in achieving superior antibacterial responses across multiple bacterial strains.

The use of mixture design methodology has become increasingly popular among researchers in various fields, particularly for creating essential oil (EO) blends. For instance, Benkhaira et al. [76], demonstrated the synergistic effects of EOs from *Ruta montana* L., *Clinopodium nepeta* (L.) Kuntze., and *Dittrichia viscosa* (L.) Greuter against *S. aureus*, and *P. aeruginosa* as innovative antiadhesive agents for 3D-printed materials. Similarly, Kachkoul et al. [77], highlighted the synergy of EOs from *Rosmarinus officinalis* L., *Eucalyptus camaldulensis* Dehnh., and *Mentha pulegium* L. against bacteria associated with lithiasis infection. Ouedrhiri et al. [78], have also reported enhanced antibacterial activity against *Bacillus subtilis*, and *S. aureus* using a ternary mixture of *Origanum compactum* Benth., *Origanum majorana* L., and *Thymus serpyllum* L.

The mixtures studied included optimal formulations featuring two predominant classes of compounds: oxygenated monoterpenes (thymol, camphor, linalool, 1,8-cineole, and 3-thujanone) and sesquiterpene hydrocarbons (α-himachalene, β-himachalene, and caryophyllene). These bioactive components target different sites within bacterial cells [79]. While hydrocarbon monoterpenes display comparatively weaker antibacterial effects, oxygenated terpenoids—containing hydroxyl (-OH) groups—exhibit stronger antibacterial properties [80]. Key compounds in the EOs, such as thymol and linalool, are known to disrupt membrane permeability by interacting with phospholipids, resulting in a fluidifying effect [58,81]. Thymol, in particular, disrupts bacterial citrate metabolism and inhibits ATP-synthesizing enzymes [82,83]. Furthermore, Burt [28], suggested that the synergistic or additive effects among EOs may stem from interactions between major and minor components. Numerous studies in the literature have investigated the synergistic antibacterial properties of EOs derived from various plants, as well as the interactions among their individual components [76,78,84,85].

Our research supports the effectiveness of combined natural antibacterial therapies in controlling pathogenic bacteria. However, to our knowledge, no prior study has demonstrated the synergistic activity of these EOs against *S. aureus*, *E. coli*, and *P. aeruginosa*.

### 2.10. Experimental Verification of the Assumed Model

Table 6 provides a comprehensive assessment of cubic models used to evaluate the antibacterial effects of essential oil (EO) combinations from *T. satureioides*, *L. angustifolia*, and *O. majorana*. This analysis is crucial for verifying the accuracy of these models in predicting antibacterial activities against three bacterial strains. The model’s reliability is supported by the close alignment of experimental results with predicted outcomes, demonstrating their strong correlation and confirming the model’s effectiveness in practical applications.

In particular, for the combination containing 76% *T. satureioides*, and 24% *O. majorana*, the experimental MIC*_E. coli_* value was recorded at 0.10 ± 0.00% (*v*/*v*), which closely aligns with the predicted value of 0.097 ± 0.00% (*v*/*v*). A mixture of 61% *T. satureioides*, 29% *L. angustifolia*, and 10% *O. majorana* showed an experimental MIC*_S.aureus_* value of 0.06 ± 0.00% (*v*/*v*), closely matching the predicted value of 0.058 ± 0.00% (*v*/*v*). Additionally, the experimental MIC*_P. aeruginosa_* for a mixture of 81% *T. satureioides*, and 19% *O. majorana* was recorded at 0.25 ± 0.00% (*v*/*v*), precisely matching the predicted value of 0.25 ± 0.00% (*v*/*v*). These findings underscore the model’s capability to accurately forecast the antibacterial potential of these EO combinations under the conditions tested.

The validation of these results is essential, as it not only verifies the robustness of the modeling approach but also enhances the understanding of how specific EO proportions can synergistically improve antibacterial effects.

## 3. Materials and Methods

### 3.1. Plant Material and Extraction of EOs

Leaves of *Thymus satureioides* Coss., *Lavandula angustifolia* Mill., and *Origanum majorana* L. were harvested from the local experimental station at the Faculty of Sciences, University Mohammed the First, Oujda, located in northeastern Morocco (34° 39′ 7.562″ N, 1° 54′ 0.812″ W). Prof. Dr. Mohamed ADDI from the same faculty authenticated the plant species. Exsiccates of the plant materials were prepared and deposited in the herbarium of the faculty, under voucher numbers PLC-23 (*T. satureioides*), PLC-24 (*L. angustifolia*), and PLC-25 (*O. majorana*). The study was conducted in compliance with applicable guidelines and regulations. Plant samples were air-dried in a shaded environment to prevent degradation of active compounds and then subjected to hydro-distillation using a Clevenger-type apparatus for 180 min, starting with 100 g of dried plant material. The extracted EOs were subsequently dried using anhydrous sodium sulfate, filtered, and preserved at 4 °C for additional analysis [14].

### 3.2. GC-MS Analysis of EOs

The chemical profiles of the three EOs were analyzed using gas chromatography (GC) coupled with a Shimadzu GC/MS-QP2010 series mass spectrometer (Shimadzu, Tokyo, Japan) [86,87,88]. The samples were vaporized and introduced via a split/splitless injector into a BP-X25 capillary column (30 m × 0.25 mm) with a non-polar stationary phase consisting of 95% dimethylpolysiloxane and 5% phenyl. Helium was used as the carrier gas at a flow rate of 3 mL/min. The injector, ion source, and interface temperatures were maintained at 250 °C. The column oven’s temperature program began at 50 °C (held for one minute), then increased to 250 °C at a rate of 10 °C/min, and was held at 250 °C for one minute.

Electron Ionization (EI) mode at 70 eV was employed to ionize the sample components, with mass scanning conducted in the range of 40–300 m/z. The mass spectrometer facilitated the separation and identification of compounds, which were matched to standards and spectral databases, including the National Institute of Standards and Technology (NIST). All data acquisition and analysis were performed using LabSolutions software (version 2.5).

### 3.3. Antioxidant Assays

#### 3.3.1. DPPH Radical Scavenging Assay

The antiradical activity of three EOs and their various combinations, generated using an experimental design approach, was assessed using 2,2-diphenyl 1-picrylhydrazyl (DPPH). This method evaluates the ability of antioxidants in the EOs to scavenge the stable DPPH free radical, which changes color from purple to yellow upon reduction. A modified protocol based on Elbouzidi et al. (2024) was used [14]. In this assay, 50 µL of the essential oil sample at different concentrations was mixed with 950 µL of a 0.1 mM DPPH methanolic solution. The mixture was incubated in the dark at room temperature for 30 min to allow the reaction to reach completion. The absorbance was then measured at 517 nm. Butylated hydroxytoluene (BHT) and ascorbic acid (AA) served as references. The radical scavenging activity (RSA) of the EOs was calculated using Equation (4), and the IC_50_ was then determined as follows:

(4) $$RSA\left(\%\right)=\left[\left(\frac{A_{b}-A_{x}}{A_{b}}\right)\right]\times 100$$

where A_b_ is the absorbance of the blank (DPPH solution without sample), and A_x_ is the absorbance of the sample.

#### 3.3.2. ABTS Radical Scavenging Activity

The ABTS radical scavenging assay was performed based on the methodologies outlined by El Hachlafi et al. (2023) [55], with minor adjustments to suit the experimental conditions. This assay measures the antioxidant activity of samples by evaluating their ability to neutralize the ABTS radical cation (ABTS^+^), which produces a blue-green chromophore with a maximum absorbance at 734 nm. The ABTS^+^ solution was prepared by mixing 7 mM ABTS stock solution with 2.45 mM potassium persulfate and incubating the mixture in the dark at room temperature for 12–16 h to allow for radical generation.

The results are presented as IC_50_ values (μg/mL), indicating the concentration required to inhibit 50% of the radicals, along with the standard deviation (±SD) from three independent replicates (*n* = 3). For comparison and validation, butylated hydroxytoluene (BHT) and ascorbic acid were utilized as standard reference antioxidants. These modifications ensured optimal conditions for precise and reliable measurement of scavenging activity.

### 3.4. Antibacterial Assays

#### 3.4.1. Strains Culture

To evaluate the antibacterial capabilities of TSEO, LAEO, and OMEO, three bacterial strains were utilized in this study, including one gram-positive bacterium: *Staphylococcus aureus* ATCC 6538, and two gram-negative bacteria: *Pseudomonas aeruginosa* ATCC 15442, and *Escherichia coli* ATCC 10536. These strains were provided by Prof. Dr. Abdeslam ASEHRAOU. Bacterial cultures were reactivated by streaking a looped needle with the culture across Luria–Bertani broth agar media. Subsequently, these cultures were incubated at 37 °C for 20 h. Fresh cultures were prepared into bacterial suspensions in 10 mL of sterile physiological NaCl solution, with turbidity calibrated to a 0.5 McFarland standard. A final bacterial density of approximately 10^6^ CFU/mL was employed for testing, adhering to the standards of National Committee for Clinical Laboratory Standards (the guidelines M07-A10), USA [89].

#### 3.4.2. Disc Diffusion Method

The antibacterial activity of the three EOs was initially tested using a modified agar disc diffusion method. The prepared culture suspensions were spread onto Mueller–Hinton (M-H) agar plates. Each sterile paper disc (6 mm) was impregnated with 8 μL of the pure oils before placement on the agar. Chloramphenicol and vancomycin served as a control (serial dilutions ranging from 32 to 0.060% (*v*/*v*)). Following a 24 h incubation period at 37 °C, the diameters of the inhibition zones were measured in millimeters, with results expressed as the mean ± SD from three independent experiments.

#### 3.4.3. Determination of MIC

The minimum inhibitory concentrations (MICs) of TSEO, LAEO, and OMEO were determined using a modified protocol by El Hachlafi et al. [90]. Samples were prepared in two-fold serial dilutions ranging from 16 to 0.060% (*v*/*v*). The EOs were mixed in M-H broth medium containing 0.15% agar and then transferred to sterile 96-well plates, with 50 μL of each dilution added per well. Additionally, 50 μL of the pre-adjusted bacterial suspensions were introduced into each well. M-H broth medium containing 0.15% agar was utilized as the growth control. After a 24 h incubation at 37 °C, 12 μL of resazurin (0.017%) was added to each well to indicate growth. The MIC was defined as the highest dilution of oil that resulted in the reduction of the blue dye resazurin to pink resorufin. All tests were conducted in triplicate.

### 3.5. Experimental Design

#### 3.5.1. Mixture Design

An augmented simplex–centroid design was utilized to optimize the antibacterial efficacy of the combined EOs from *T. satureioides*, *L. angustifolia*, and *O. majorana*, following the methodology described by Benkhaira et al. [76]. This experimental design enables the systematic exploration of mixture proportions to identify the most effective combination. The composition details of the EO system are provided in Table 1. Each essential oil in the mixture was allowed to vary within a range of 0 to 1, with the sum of the proportions of the three oils always equaling 1, as specified in Table 7.

The antibacterial activity of these EO mixtures was assessed against three clinically significant pathogens, providing insights into their synergistic or individual effects in inhibiting microbial growth. This approach not only ensures precise formulation but also highlights the potential of EO combinations in addressing bacterial challenges effectively.

#### 3.5.2. Experimental Matrix and Mathematical Model

**Figure 12:**
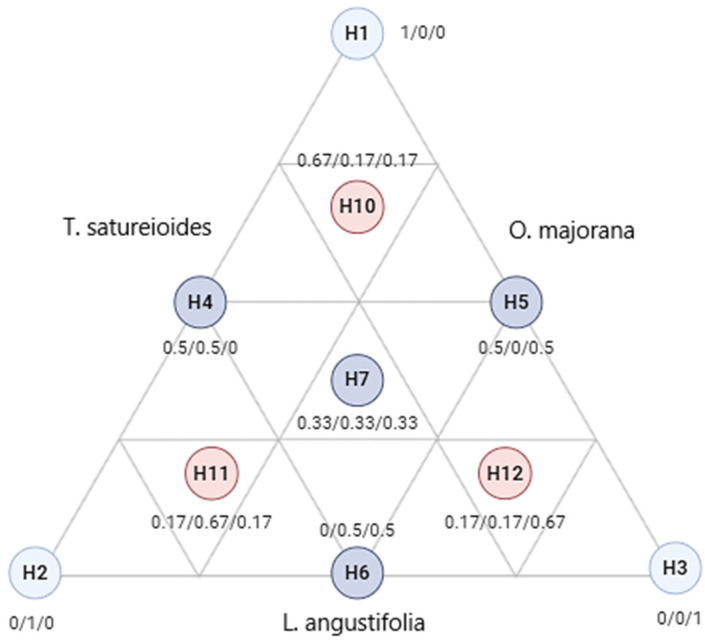
Equilateral triangle of the arrangement of mixtures using the simplex centroid design method. H1: *T. satureioides* EO; H2: *L. angustifolia* EO; H3: *O. majorana* EO.

In this study, 10 experimental trials were designed and represented within an equilateral triangle (Figure 12). This triangular design illustrates the different proportions of the components under investigation. The three pure components are positioned at the vertices of the triangle (H1, H2, H3). Binary mixtures, where the components are combined in equal proportions (0.5/0.5), are located at the midpoints of the triangle’s edges (H4, H5, H6). A ternary mixture in equal proportions (0.33/0.33/0.33) is positioned at the triangle’s centroid (H7).

To ensure consistency and validate the results, the experiment was repeated three times, incorporating three control points (H10, H11, H12) that represent ternary mixtures with varying proportions (0.67/0.16/0.16). A cubic model was employed to describe the responses, taking into account the independent variables. This approach allowed for a comprehensive analysis of the interactions between components, with the responses expressed through the following mathematical equation.

$$Y={\alpha_{1}H}_{1}+{\alpha_{2}H}_{2}+{\alpha_{3}H}_{3}+\alpha_{12}H_{1}H_{2}+\alpha_{13}H_{1}H_{3}+\alpha_{23}H_{2}H_{3}+\alpha_{123}H_{1}H_{2}H_{3}+ɛ$$

In the model, Y represents the experimental response, quantified as the MIC (%, *v*/*v*). The coefficients α_1_, α_2_ and α_3_ correspond to the linear effects of the individual components. The binary interaction effects between pairs of components are represented by the coefficients α_12_, α_13_, and α_23_, while α_123_ captures the interaction effect of the ternary combination. The term ε (epsilon) accounts for the regression error, representing the variation not explained by the model. This formulation allows for the quantification of both individual and interactive contributions of the components to the overall response.

### 3.6. Statistical Analysis and Optimisation Tools

The model’s adequacy was assessed by comparing the mean square lack of fit to the mean square pure error (MSLOF/MSPE), where higher values may signal potential inadequacies. The model’s quality was also evaluated using the coefficient of determination (R^2^). The statistical validity of the mathematical model was examined at a 95% confidence level using the F-ratio, calculated as the ratio of the mean square regression (MSR) to the mean square residual (MSr). A higher F-value suggests greater variability explained by the model [90]. Additionally, the model’s adequacy was tested by analyzing the ratio of the mean square lack of fit (MSLOF) to the mean square pure error (MSPE), where elevated values may indicate potential issues with the model’s fit. The coefficient of determination R^2^ was also employed to assess the overall quality of the model, reflecting its ability to explain variability in the data.

The significance of the individual factors within the model was evaluated using the Student’s *t*-test, while the overall model significance was confirmed through the F-test in an analysis of variance (ANOVA). Statistical analyses were conducted using Design-Expert software (version 12) and SAS JMP^®^ (version 14), with results expressed as means ± standard deviation (SD) from three independent replicates (n = 3).

For optimization purposes, contour and 3D surface plots were utilized to visually represent trade-off regions among the studied components, facilitating the identification of optimal mixtures. The desirability function was applied to pinpoint the best outcomes, balancing the factors to achieve the most favorable results. This function adjusts the model within a range of 0 to 1, where 0 represents an undesirable outcome and 1 reflects a highly desirable one, ensuring practical and efficient optimization of the system.

## 4. Conclusions

This study effectively optimized the antibacterial and antioxidant properties of essential oil (EO) mixtures from *T. satureioides*, *L. angustifolia*, and *O. majorana* using a simplex–centroid design. Chemical analysis revealed that TSEO was predominantly composed of β-himachalene (42.16%), α-himachalene (20.04%), and caryophyllene (10.80%), while LAEO contained mainly linalool (19.12%), 1,8-cineol (15.42%), and thymol (12.57%). OMEO was characterized by high levels of camphor (39.58%), 3-thujanone (23.03%), and 1,8-Cineol (7.42%).

The resulting formulations demonstrated significant antibacterial activity, with minimum inhibitory concentrations (MIC) of 0.097% (*v*/*v*) against *E. coli*, 0.058% (*v*/*v*) against *S. aureus*, and 0.250% (*v*/*v*) against *P. aeruginosa*. The optimized mixture, consisting of 76% *T. satureioides*, and 24% *O. majorana*, achieved a desirability of 99.99%, showcasing broad-spectrum antibacterial efficacy. In antioxidant evaluations, *L. angustifolia* EO exhibited superior performance, with IC_50_ values of 84.36 µg/mL, and 139.61 µg/mL in DPPH and ABTS assays, respectively. This performance surpasses that of *T. satureioides*, and *O. majorana*, underscoring its potential in mitigating oxidative stress.

These results suggest promising applications for these EO formulations across various industries. In healthcare, they may serve as natural alternatives or adjuncts to conventional antibiotics, especially against multidrug-resistant pathogens. In the food industry, these formulations could act as natural preservatives, extending shelf life and enhancing safety. Additionally, their potent antioxidant properties position them as potential candidates for incorporation into cosmetic products to combat oxidative damage and aging. However, several limitations must be considered before scaling up these formulations for commercial use. The stability of the essential oil mixtures may be influenced by factors such as temperature, light, and storage conditions, which could affect their efficacy over time. Furthermore, the cost of sourcing and processing these essential oils may limit their widespread use in large-scale applications. Developing more cost-effective methods for extraction and ensuring long-term stability through formulation adjustments or controlled-release systems are essential steps to enhance their practical applicability.

## Figures and Tables

**Table 1:** Phytochemical profile of *T. satureioides*, *L. angustifolia*, and *O. majorana* EOs using GC-MS.

Compound *	Area (%)			Retention Index (RI)		Identification
	TSEO	LAEO	OMEO	RI_Calc._ **	RI_Lit._ ***	
Camphene	-	-	0.49	944	944	RI, MS
β-Pinene	-	0.27	-	980	980	RI, MS
β-Myrcene	-	0.59	0.47	990	993	RI, MS
*o*-Cymene	-	0.96	0.39	1013	1015	RI, MS
*p*-Cimene	0.36	-	-	1016	1017	RI, MS
D-Limonene	-	-	0.58	1024	1024	RI, MS
1,8-Cineol	0.50	**15.42**	**7.42**	1022	1020	RI, MS
Ocimene	-	0.58	-	1034	1035	RI, MS
γ-Terpinene	0.62	1.91	0.22	1052	1052	RI, MS
*trans*-Linalool oxide	-	1.00	-	1066	1067	RI, MS
*cis*-Linalool Oxide	-	0.95	-	1084	1084	RI, MS
Linalool	0.72	**19.12**	0.80	1095	1104	RI, MS
3-Thujanone	-	-	**23.03**	1090	1093	RI, MS
Thujone	-	-	5.69	1097	1097	RI, MS
Arthole	0.56	-	-	1110	-	MS
Camphor	-	10.02	**39.58**	1122	1122	RI, MS
*cis*-*p*-Menthan-3-one	-	0.13	-	1149	1152	RI, MS
*p*-Menthanone	-	1.70	-	1148	1148	RI, MS
Borneol	-	5.66	1.58	1150	1148	RI, MS
4-Terpineol	-	2.99	2.26	1175	1175	RI, MS
Neoisomenthol	-	1.28	-	1165	1165	RI, MS
*p*-Menth-1-en-8-ol	-	5.32	0.62	1168	1168	RI, MS
Verbenone	-	0.66	-	1207	1206	RI, MS
Pulegone	-	4.31	0.46	1233	1233	RI, MS
Linalool acetate	-	5.57	-	1254	1254	RI, MS
*cis*-Farnesol	-	1.90	-	1722	1722	RI, MS
Geraniol acetate	-	0.44	-	1750	1750	RI, MS
Carvacrol	2.79	3.26	1.27	1318	1317	RI, MS
Thymol	9.71	**12.57**	4.00	1298	1297	RI, MS
Nerol acetate	-	0.42	-	1722	1722	RI, MS
Linalyl iso-valerate	-	0.70	-	1580	1579	RI, MS
α-Gurjunene	0.81	-	-	1409	1407	RI, MS
D-Longifolene	0.70	-	-	1411	-	MS
Caryophyllene	**10.80**	0.21	0.38	1415	1416	RI, MS
α-Longipinene	0.66	-	-	1454	-	MS
α-Himachalene	**20.04**	-	0.78	1476	1476	RI, MS
Mansonone C	0.75	-	-	1510	-	MS
δ-Cadinene	1.45	-	-	1518	1518	RI, MS
Laurene	1.38	-	-	1530	-	MS
α-Calacorene	0.56	-	-	1567	-	MS
Caryophyllene oxide	-	0.69	1.23	1580	1580	RI, MS
Epiglobulol	-	-	5.50	1583	1582	RI, MS
*cis*-Limonene oxide	-	-	0.66	1598	-	MS
β-Himachalenoxide	1.14	-	-	1610	-	MS
τ-Cadinol	-	0.79	-	1638	1638	RI, MS
α-Bisabolol	-	0.58	-	1645	-	MS
Tumerone	1.19	-	-	1666	1665	RI, MS
Germacrone	1.10	-	-	1693	1693	RI, MS
α-Cedrene	2.00	-		1421	1422	RI, MS
β-Himachalene	**42.16**	-	2.10	1523	1520	RI, MS
α-Humulene	-	-	0.49	1455	1455	RI, MS
**MH**	65.35	3.73	5.03			
**OM**	14.28	94.00	86.71			
**SH**	14.74	0.21	0.38			
**OS**	5.63	2.06	7.88			
**Total**	**100**	**100**	**100**			

* Components were identified using mass spectrometry (MS) and retention indices (RI). ** The Kovats index was calculated using an alkane series (C8–C24) on a capillary column. *** Kovats indices (retention indices) were referenced from established data libraries, including NIST. The essential oils’ constituents are categorized into four primary groups: monoterpene hydrocarbons (MH), oxygenated monoterpenes (OM), sesquiterpene hydrocarbons (SH), and oxygenated sesquiterpenes (OS). The major components within each oil are highlighted in bold for emphasis.

**Table 2:** Inhibition zones diameters (IZ) and MIC values of the studied EOs compared to conventional antibiotics.

	*E. coli*	*S. aureus*	*P. aeruginosa*
	IZ (mm ± SD) *		
TSEO	26.80 ± 0.30	35.05 ± 1.95	30.25 ± 0.75
LAEO	12.70 ± 1.54	19.75 ± 1.75	15.50 ± 3.00
OMEO	3.56 ± 0.10	1.64 ± 0.50	6.42 ± 0.75
Vancomycin	20.65 ± 0.30	28.68 ± 1.10	21.15 ± 1.64
Chloramphenicol	26.90 ± 0.55	34.23 ± 0.80	33.35 ± 0.56
	**MIC (*v*/*v* %)**		
TSEO	0.25	0.5	0.375
LAEO	1	2	1
OMEO	2	2	2
Vancomycin	0.25	0.5	0.5
Chloramphenicol	0.062	0.25	0.25

* Values are expressed as means ± SD of three separate experiments.

**Table 3:** Matrix of simplex centroid design and results for the antibacterial mixtures.

No. ^a^	*T. saturioides*	*L. angustifolia*	*O. majorana*	Observed Responses (MIC, % *v*/*v*) ^b^		
				*E. coli*	*S. aureus*	*P. aeruginosa*
1	1	0	0	0.25	0.5	0.375
2	0	1	0	1	2	1
3	0	0	1	2	2	2
4	0.50	0.50	0	0.25	0.25	0.5
5	0.50	0	0.50	0.375	0.5	0.5
6	0	0.50	0.50	0.5	2	1
7	0.333	0.333	0.333	0.5	0.5	0.75
8	0.333	0.333	0.333	0.5	0.5	0.75
9	0.333	0.333	0.333	0.5	0.5	0.75
10	0.667	0.167	0.167	0.25	0.25	0.5
11	0.167	0.667	0.167	0.5	0.5	1
12	0.167	0.167	0.667	0.5	1	1

^a^ The experiments were conducted following randomization. ^b^ Each test was performed in triplicate, and the results are presented as means ± SD.

**Table 4:** Variance analysis for the three fitted models.

MIC*_E. coli_*	Model	DF	SS	MS	F	*p*-Value
	R	6	2.4698155	0.411636	16.0996	0.0039 *
	r	5	0.1278408	0.025568		
	Total	11	2.5976563			
	R^2^	0.9117				
MIC*_S. aureus_*	Model	DF	SS	MS	F	*p*-Value
	R	6	5.0997736	0.849962	12.5836	0.0069 *
	r	5	0.3377264	0.067545		
	Total	11	5.4375000			
	R^2^	0.9269				
MIC*_P. aeruginosa_*	Model	DF	SS	MS	F	*p*-Value
	R	6	1.9836508	0.330608	32.0945	0.0008 *
	r	5	0.0515054	0.010301		
	Total	11	2.0351563			
	R^2^	0.9975				

* statistically significant at *p* < 0.05.

**Table 5:** Coefficients of the two presumed models and their level of significance (*p*-value).

Term	Coeff.	*E. coli*		*S. aureus*		*P. aeruginosa*	
		Estim.	*p*-Value	Estim.	*p*-Value	Estim.	*p*-Value
*T. satureioides* (Mixture)	α_1_	0.275	0.1351	0.57181	0.0717	0.38840	0.0107 *
*L. angustifolia* (Mixture)	α_2_	1.01363	0.0012 *	1.86727	0.0007 *	1.04749	0.0001 *
*O. majorana* (Mixture)	α_3_	1.91136	<0.0001 *	1.98090	0.0005 *	1.95659	<0.0001 *
T. saturioides * L. angustifolia	α_12_	−1.42272	0.1268	−4.12181	0.0224 *	−0.6281	0.2591
T. satureioides * O. majorana	α_13_	−3.12727	0.0101 *	−2.89454	0.0706	−2.81000	0.0023 *
L. angustifolia * O. majorana	α_23_	−4.15	0.0031 *	−0.30363	0.8197	−1.99181	0.0100 *
T. satureioides * L. angustifolia * O. majorana	α_123_	9.44999	0.0758	−6.48004	0.3892	6.48002	0.0606

* Bold red statistically significant at *p* < 0.05. Coeff.: coefficients.

**Table 6:** Expected and observed responses for the test point that the best-fit mixes were able to achieve.

Strains		MIC (%, *v*/*v*)	Proportions of Each EO (%)		
			*T. satureioides*	*L. angustifolia*	*O. majorana*
*E. coli*	Predi. ^a^	0.097 ± 0.000	76%	0%	24%
	Exp. ^b^	0.100 ± 0.000			
*S. aureus*	Predi.	0.058 ± 0.000	61%	29%	10%
	Exp.	0.060 ± 0.000			
*P. aeruginosa*	Predi.	0.250 ± 0.000	81%	0	19%
	Exp.	0.250 ± 0.000			

^a^ The experimental value is represented as the average of three replicates. ^b^ The expected value includes the response’s standard deviation (±SD), as determined by the model.

**Table 7:** The independent variables within the mixture.

Components	Coded Variables	Level −	Level +
*T. satureioides*	H1	0	1
*L. angustifolia*	H2	0	1
*O. majorana*	H3	0	1
Sum of proportions		1	

## Data Availability

The original contributions presented in this study are included in the article/Supplementary Material. Further inquiries can be directed to the corresponding author(s).

## Supplementary Materials

The following supporting information can be downloaded at: https://www.mdpi.com/article/10.3390/ph18010057/s1, Figure S1. TIC chromatogram of the volatile composition of *T. satureioides* EO using GC-MS. Numbers indicate compound names as in Table S1. Volatile compounds are found in the essential oil of *T. satureioides*; Figure S2. TIC chromatogram of the volatile composition of *L. angustifolia* EO using GC-MS. Numbers indicate compound names as in Table S2. Volatile compounds found in the essential oil of *L. angustifolia*; Figure S3. TIC chromatogram of the volatile composition of *O. majorana* EO using GC-MS. Numbers indicate compound names, as in Table S3. Volatile compounds are found in the essential oil of *O. majorana*.
