# Supplementary material for: Optimization of the Antibacterial Activity of a Three-Component Essential Oil Mixture from Moroccan Thymus satureioides, Lavandula angustifolia, and Origanum majorana Using a Simplex–Centroid Design
Source: Pharmaceuticals (Basel). 2025 Jan 7;18(1):57. doi: 10.3390/ph18010057 (PMC11769045; doi:10.3390/ph18010057)

Supplementary file

Optimization of the Antibacterial Activity of a Three-Component Essential Oil Mixture from *Thymus satureioides*, *Lavandula angustifolia*, and *Origanum majorana* Using a Simplex-Centroid Design

1. *Thymus satureioides* Coss. (Savory thym)

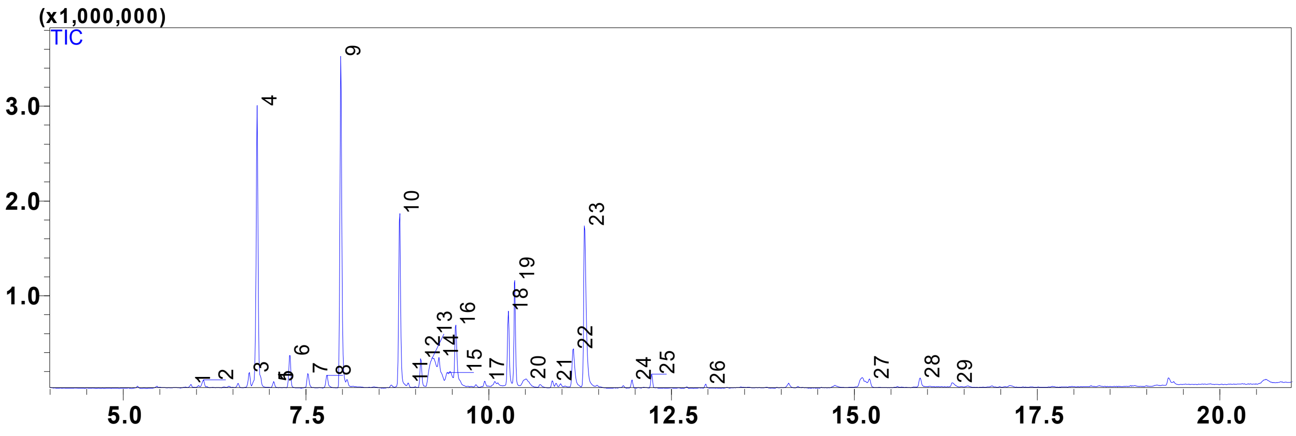

Figure S1. TIC chromatogram of the volatile composition of *T. satureioides* EO using GC-MS. Numbers indicate compounds names as in Table S1.

Table S1. Volatile compounds found in the essential oil of *T. satureioides*

No.	Compounds	R.T. (min)	Area (%)
**1**	β-Pinene	5.926	0.27
**2**	β-Myrcene	6.098	0.59
**3**	*o*-Cymene	6.722	0.96
**4**	1,8-Cineol	6.833	15.42
**5**	Ocimene	7.059	0.58
**6**	γ-Terpinene	7.279	1.91
**7**	Linalool oxide	7.527	1.00
**8**	*cis*-Linalool Oxide	7.787	0.95
**9**	Linalool	7.977	19.12
**10**	Camphor	8.780	10.02
**11**	*cis*-*p*-Menthan-3-one	8.896	0.13
**12**	*p*-Menthanone	9.069	1.70
**13**	Borneol	9.229	5.66
**14**	4-Terpineol	9.316	2.99

**15**	Neoisomenthol	9.432	1.28
**16**	*p*-menth-1-en-8-ol	9.547	5.32
**17**	Verbenone	9.945	0.66
**18**	Pulegone	10.266	4.31
**19**	Linalool acetate	10.355	5.57
**20**	*cis*-Farnesol	10.508	1.90
**21**	Geraniol acetate	10.870	0.44
**22**	Carvacrol	11.155	3.26
**23**	Thymol	11.312	12.57
**24**	Nerol acetate	11.957	0.42
**25**	Linalyl *iso*-valerate	12.226	0.70
**26**	Caryophyllene	12.965	0.21
**27**	Caryophyllene oxide	15.207	0.69
**28**	τ-Cadinol	15.899	0.79
**29**	α-Bisabolol	16.343	0.58

β-Pinene

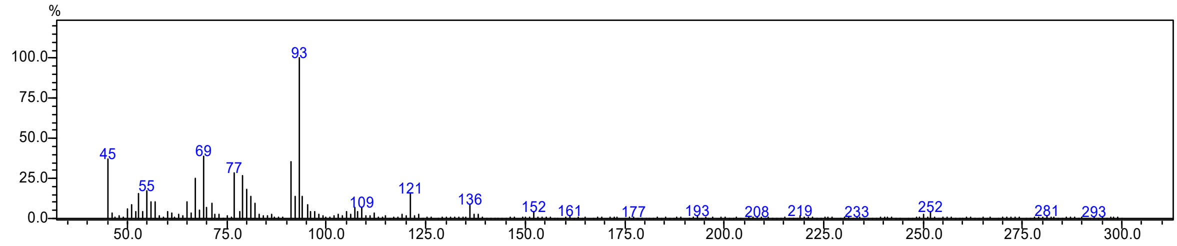

β-Myrcene

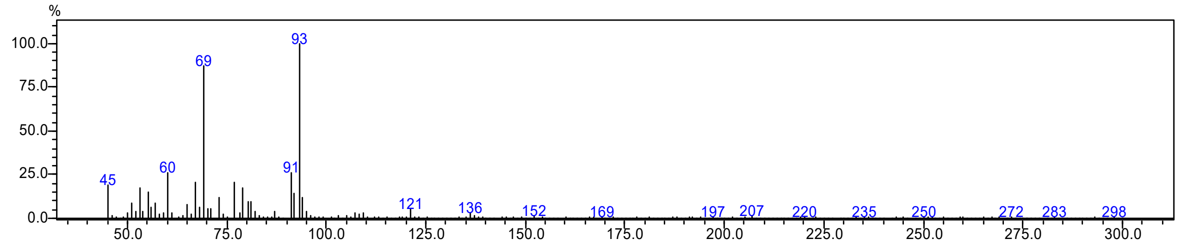

***o*-Cymene**

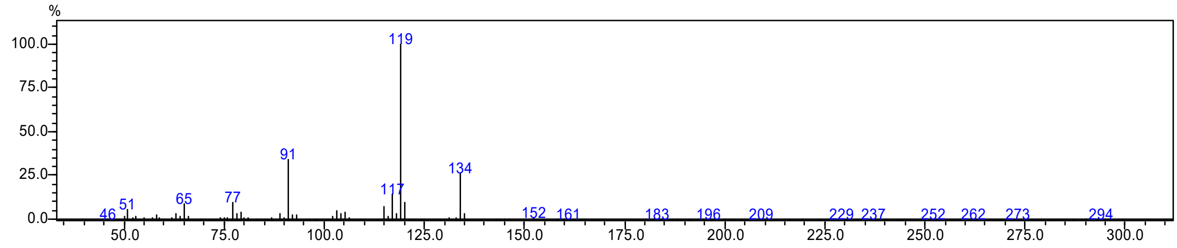

1,8-Cineol

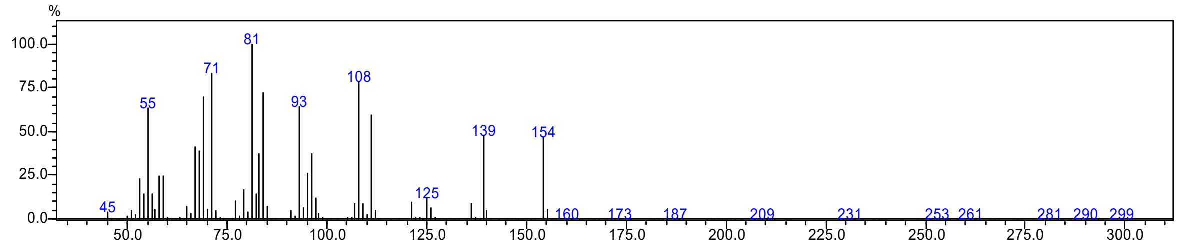

Ocimene

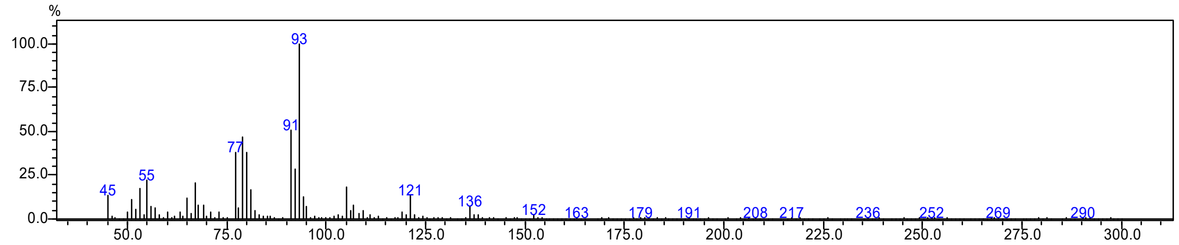

γ-Terpinene

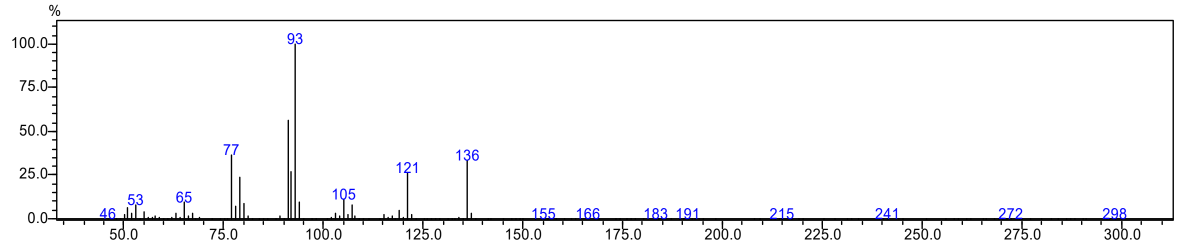

Linalool oxide

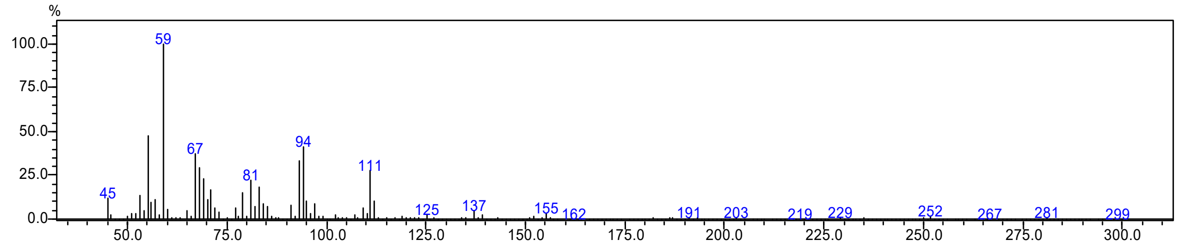

***cis*-Linalool Oxide**

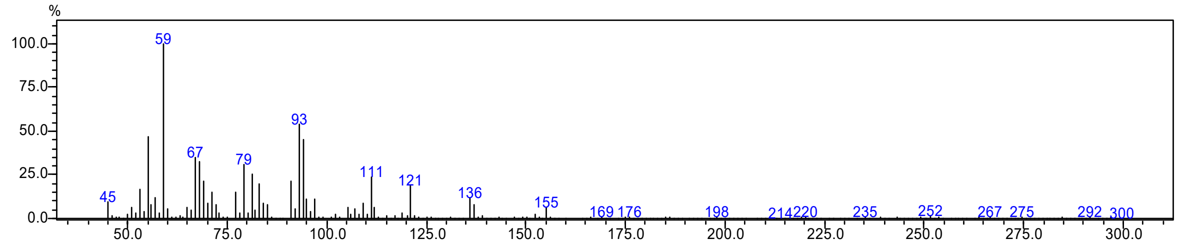

Linalool

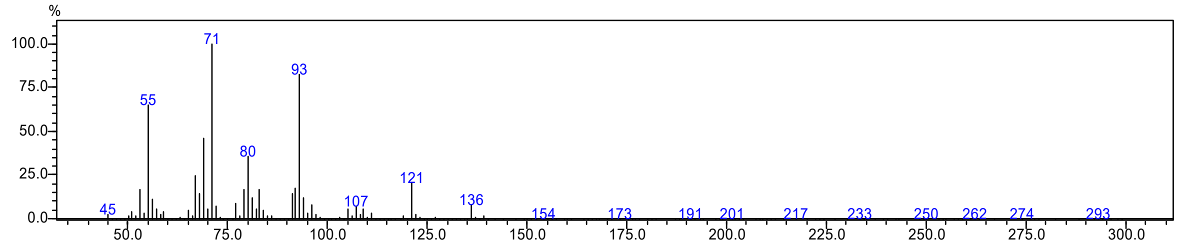

Camphor

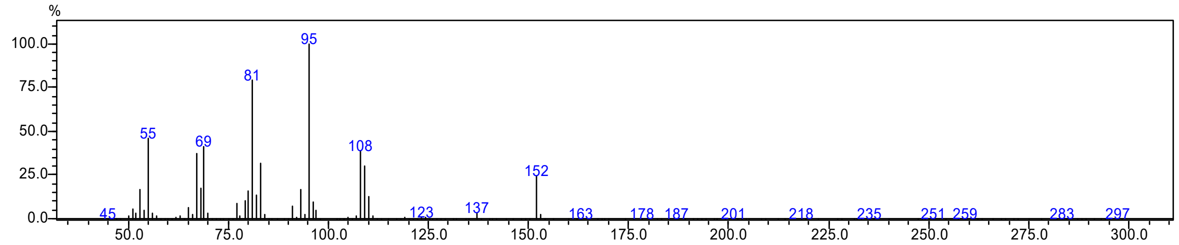

cis-p-Menthan-3-one

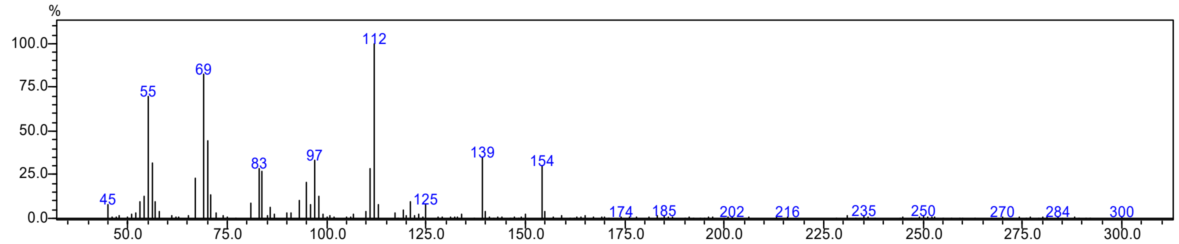

p-Menthanone

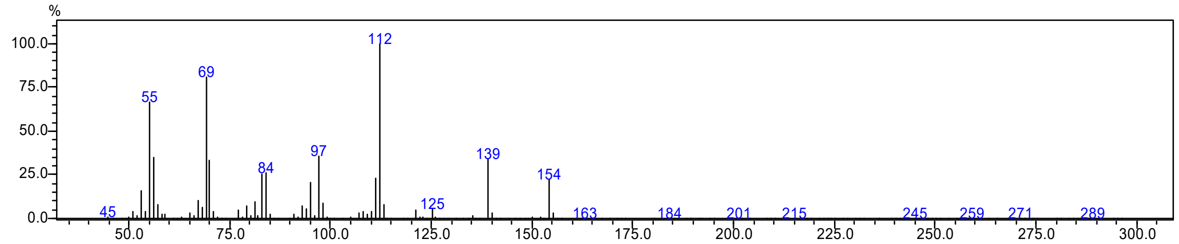

Borneol

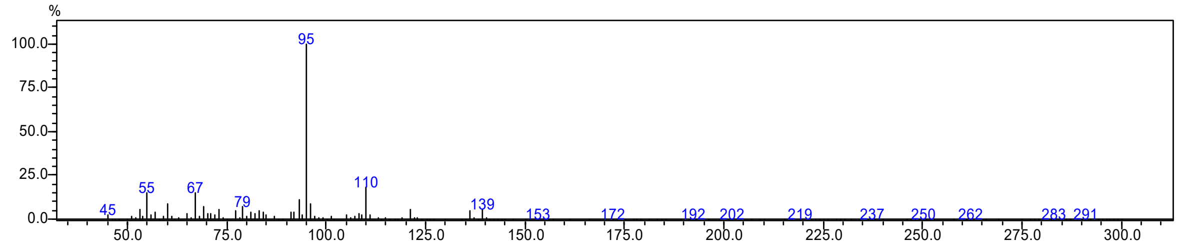

4-Terpineol

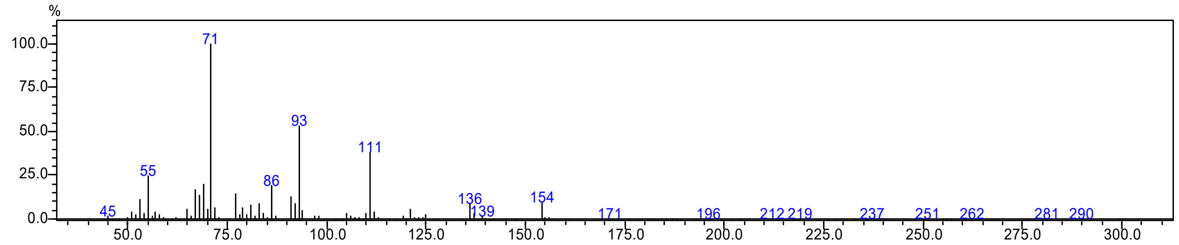

Neoisomenthol

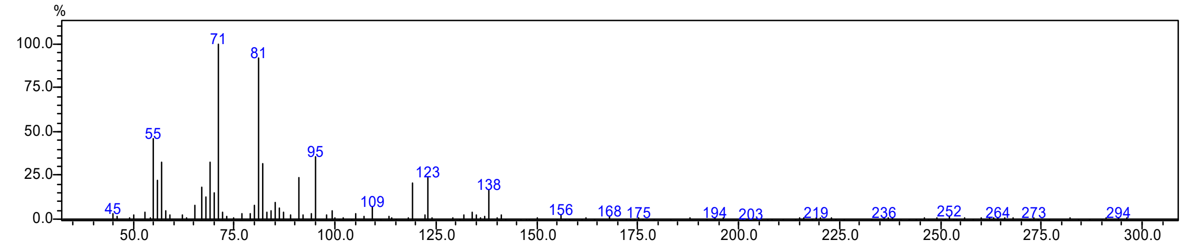

***p*-menth-1-en-8-ol**

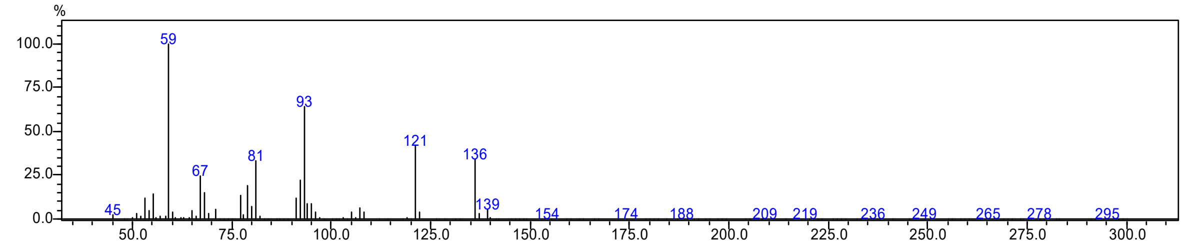

Verbenone

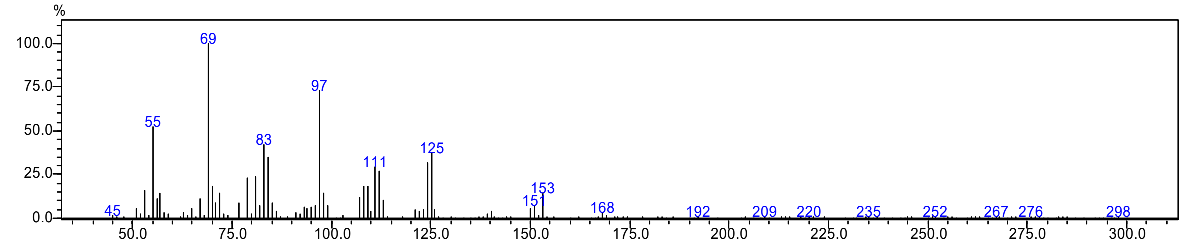

Pulegone

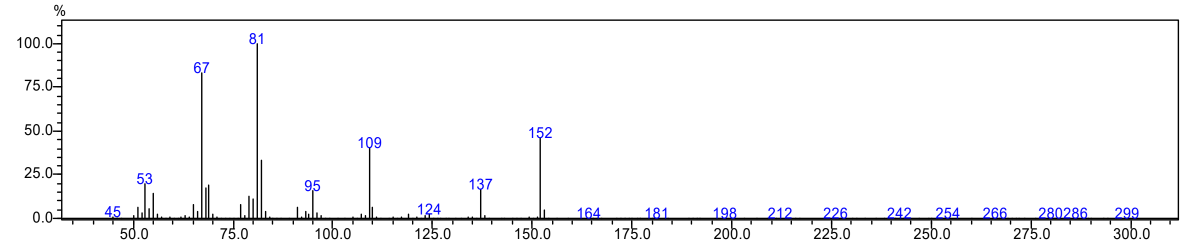

Linalool acetate

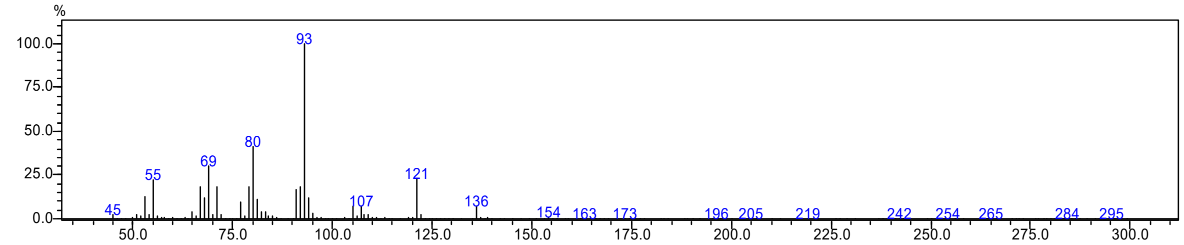

cis-Farnesol

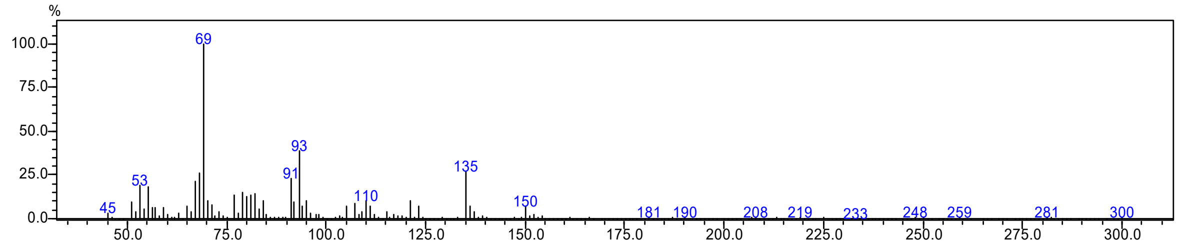

Geraniol acetate

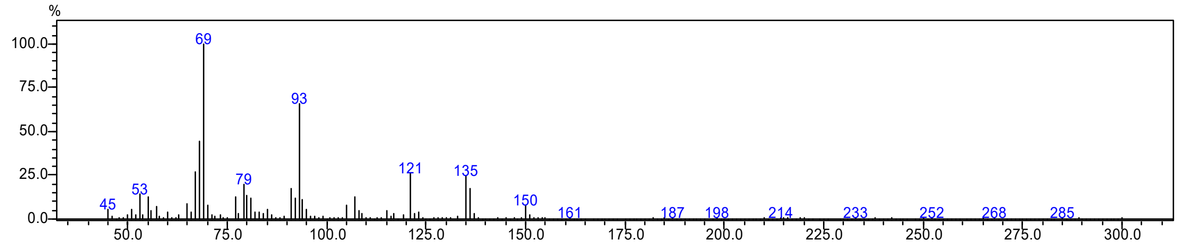

Carvacrol

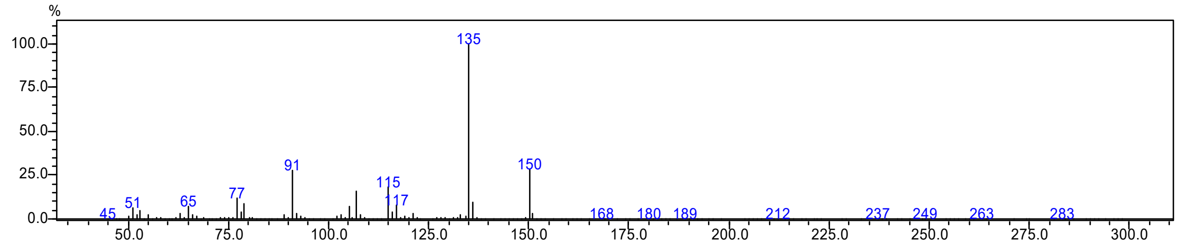

Thymol

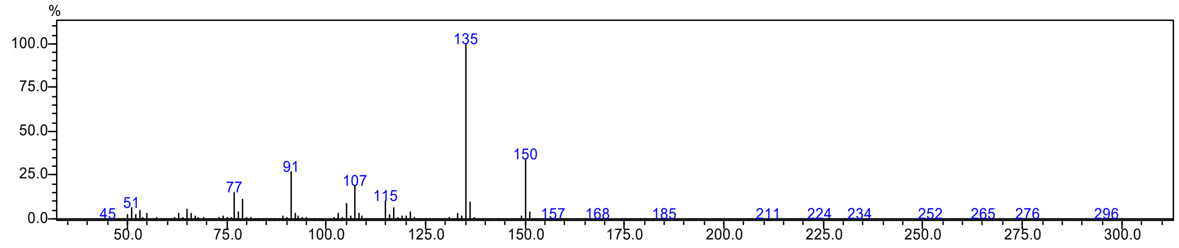

Nerol acetate

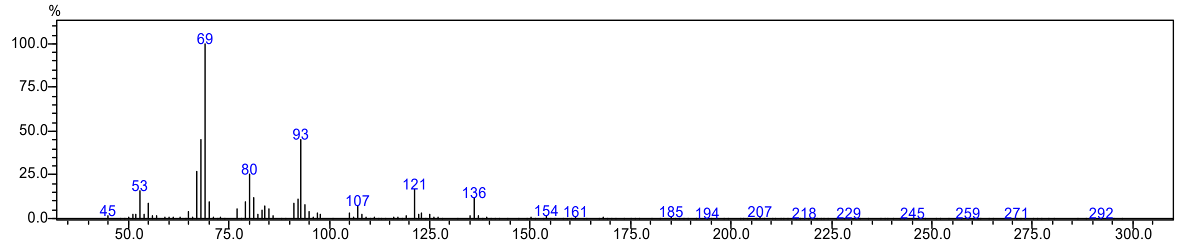

Linalyl iso-valerate

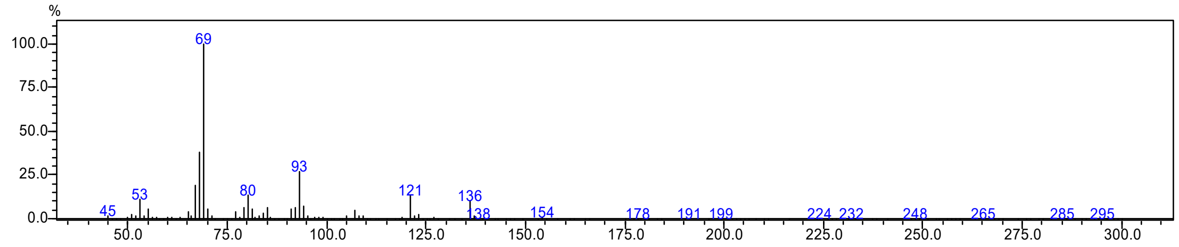

Caryophyllene

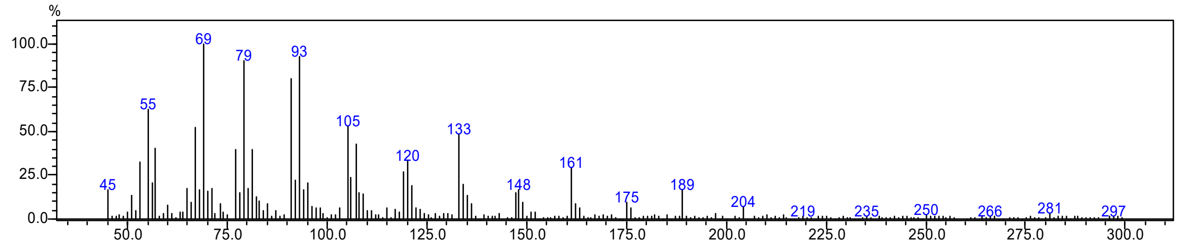

Caryophyllene oxide

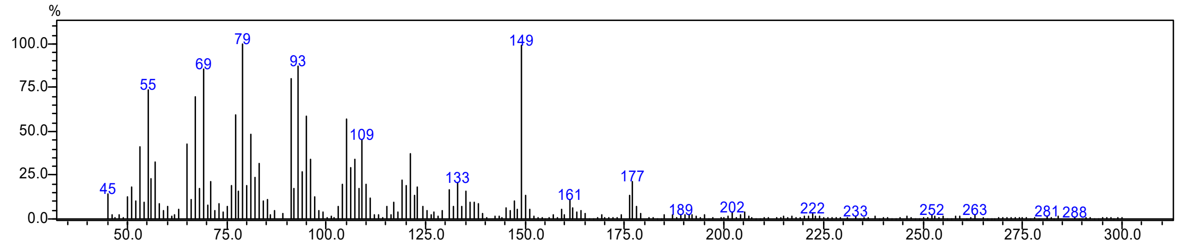

τ-Cadinol

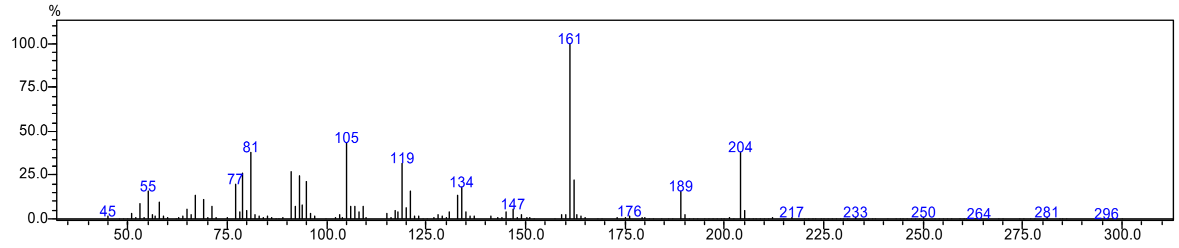

α-Bisabolol

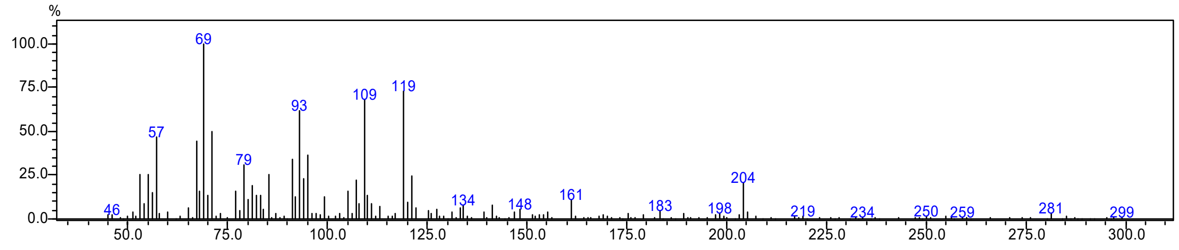

2. *Lavandula angustifolia* Mill. (lavender)

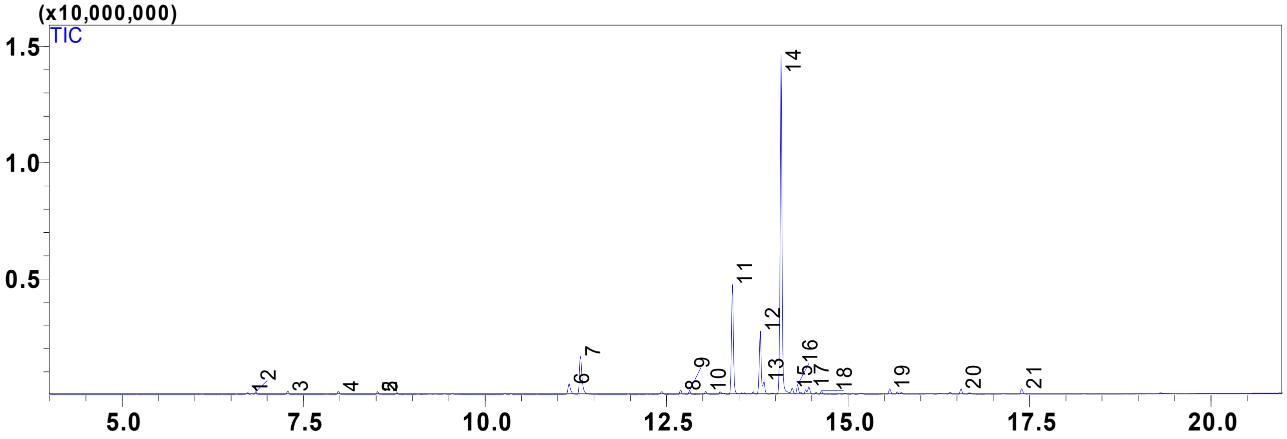

Figure S2. TIC chromatogram of the volatile composition of *L. angustifolia* EO using GC-MS. Numbers indicate compounds names as in Table S2.

Table S2. Volatile compounds found in the essential oil of *L. angustifolia.*

No.	Compounds	R.T. (min)	Area (%)
**1**	*p*-Cimene	6.727	0.36
**2**	1,8-Cineol	6.841	0.50
**3**	γ-Terpinene	7.284	0.62
**4**	Linalool	7.980	0.72
**5**	Arthole	8.519	0.56
**6**	Carvacrol	11.157	2.79
**7**	Thymol	11.315	9.71
**8**	α-Gurjunene	12.692	0.81
**9**	D-longifolene	12.818	0.70
**10**	α-Longipinene	13.040	0.66
**11**	α-Himachalene	13.406	20.04
**12**	Caryophyllene	13.790	10.80
**13**	α-Cedrene	13.839	2.00
**14**	β-Himachalene	14.077	42.16
**15**	Mansonone C	14.231	0.75
**16**	δ-Cadinene	14.305	1.45
**17**	Laurene	14.461	1.38
**18**	α-Calacorene	14.633	0.56
**19**	β-Himachalenoxide	15.573	1.14
**20**	Tumerone	16.556	1.19
**21**	Germacrone	17.392	1.10

p-Cimene

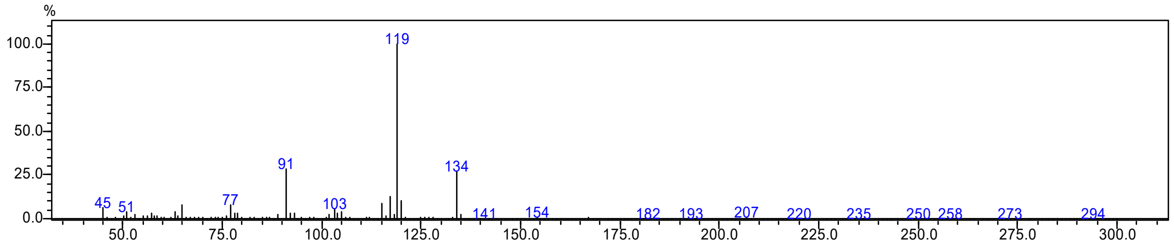

1,8-Cineol

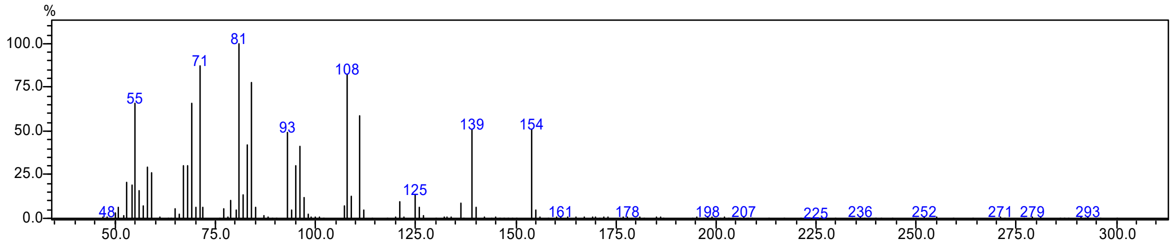

γ-Terpinene

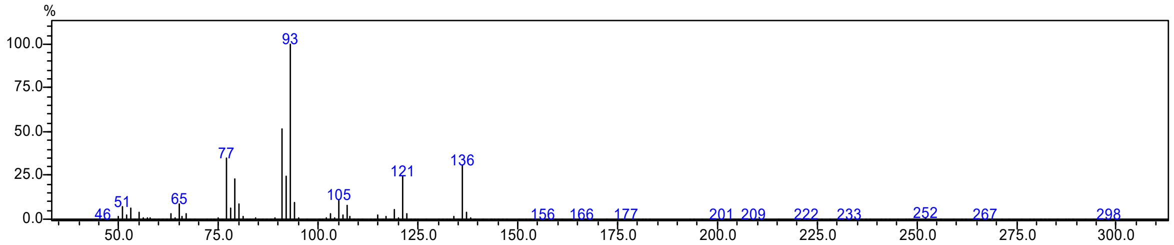

Linalool

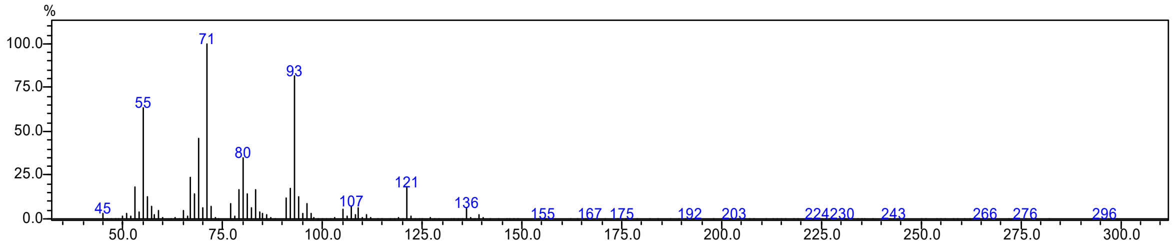

Arthole

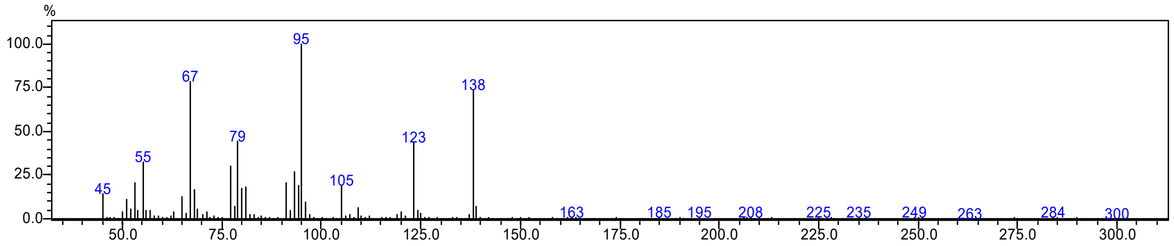

Carvacrol

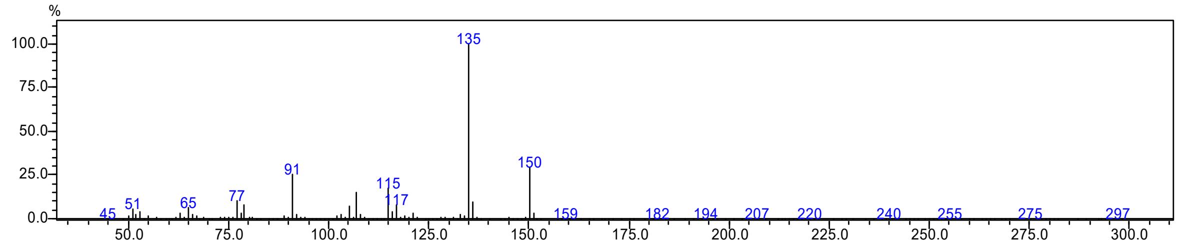

Thymol

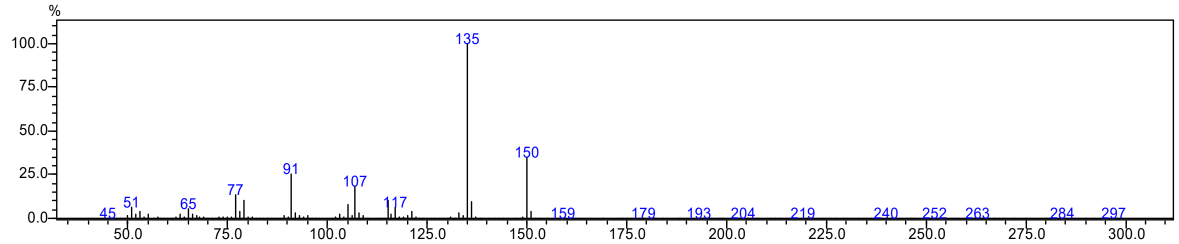

α-Gurjunene

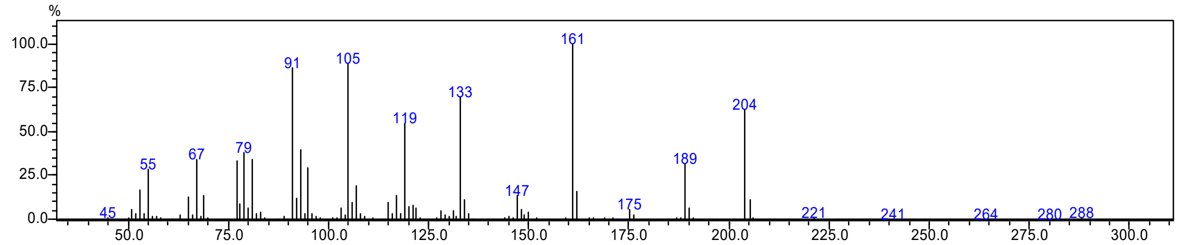

D-longifolene

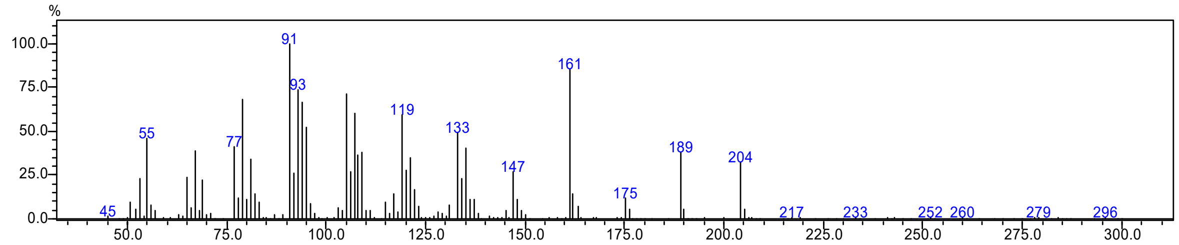

α-Longipinene

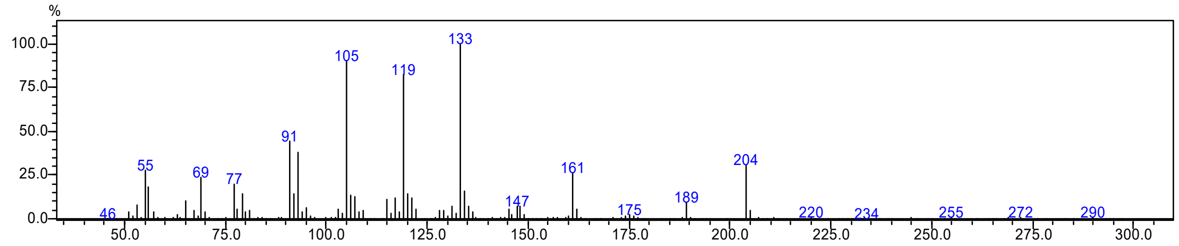

α-Himachalene

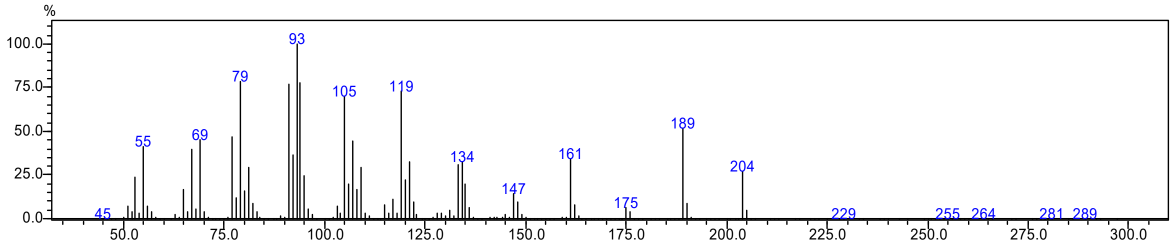

Caryophyllene

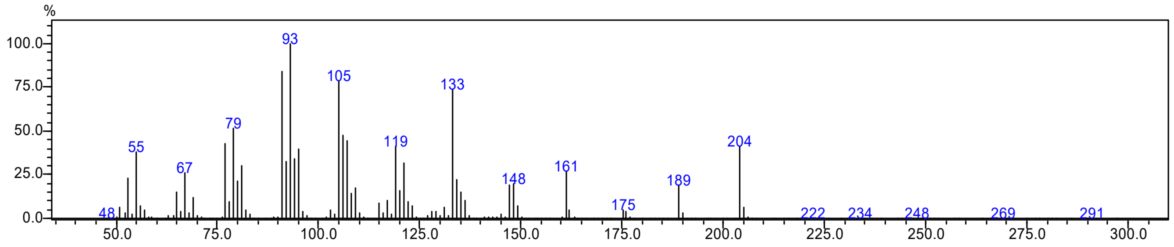

α-Cedrene

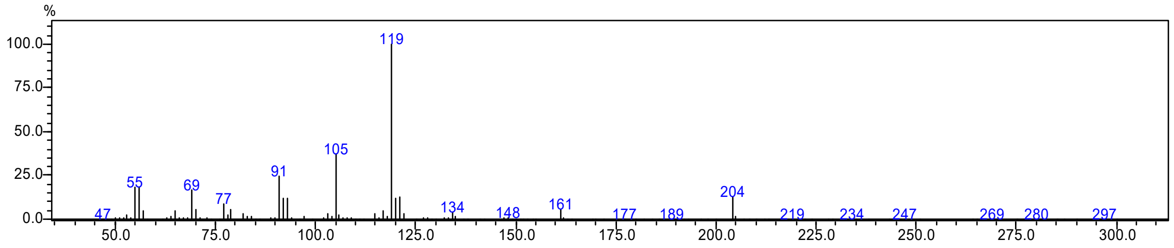

β-Himachalene

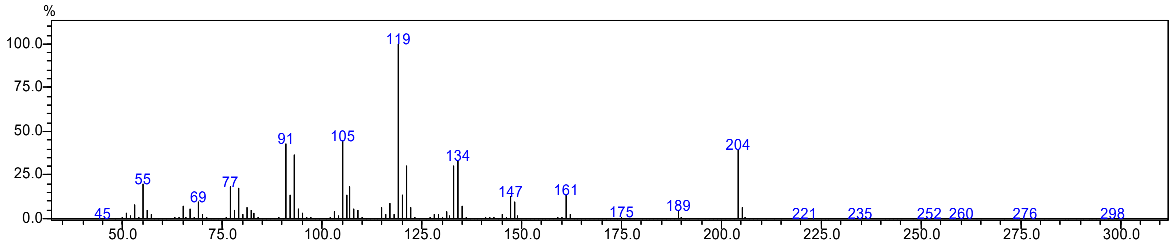

Mansonone C

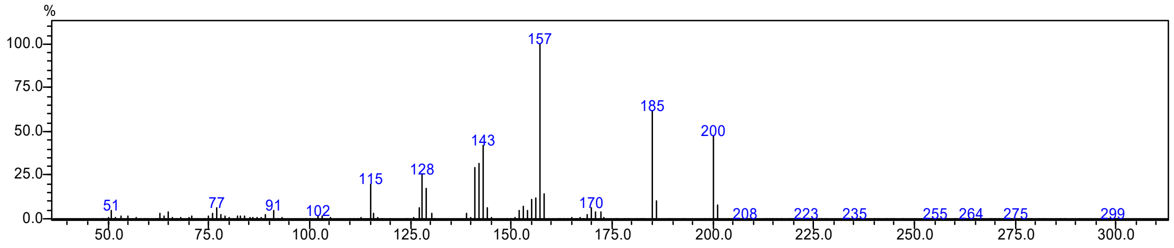

δ-Cadinene

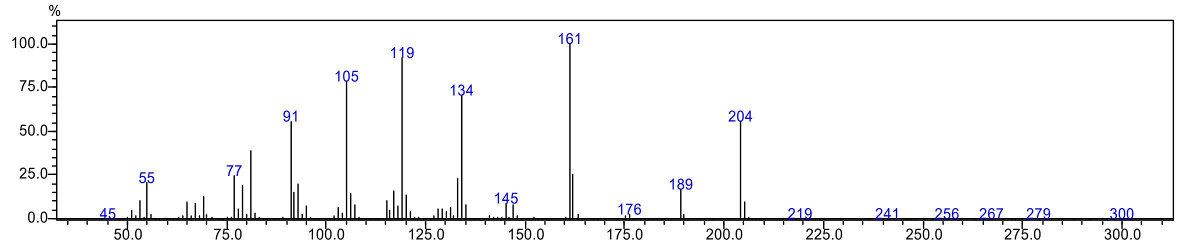

Laurene

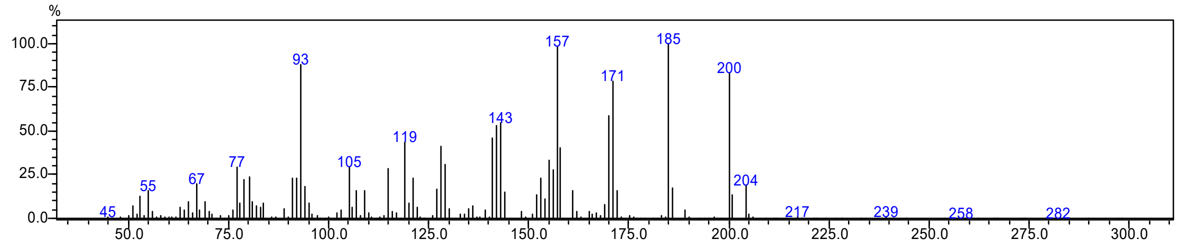

α-Calacorene

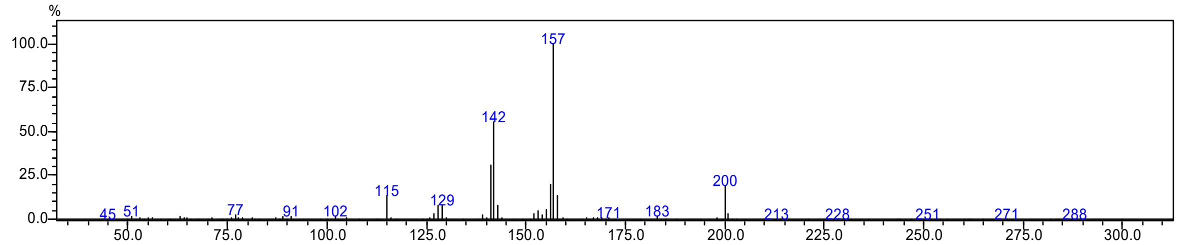

β-Himachalenoxide

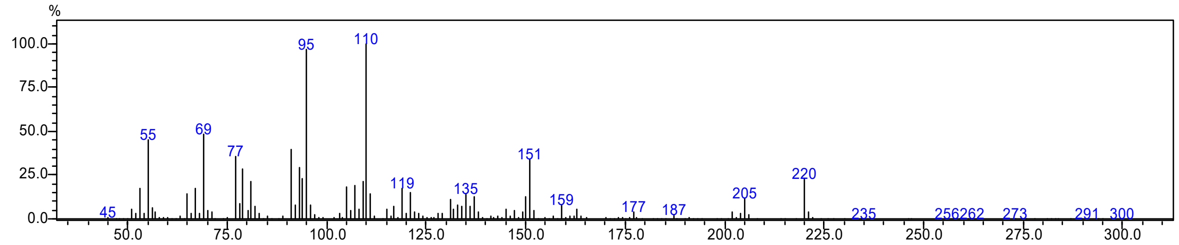

Tumerone

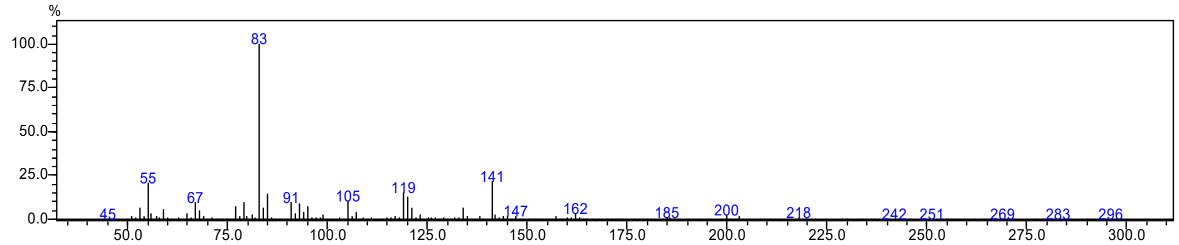

Germacrone

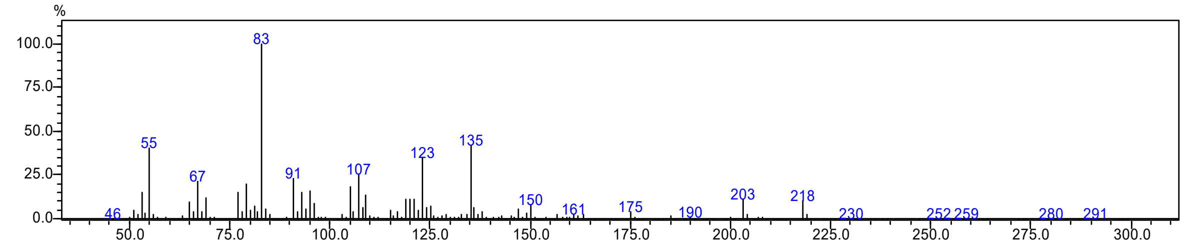

%
100.0
75.0
50.0
25.0
0.0
83
55
67
91
107
123
135
150
161
175
190
203
218
230
252
259
280
291
46
50.0
75.0
100.0
125.0
150.0
175.0
200.0
225.0
250.0
275.0
300.0

3. *Origanum majorana* L. (Sweet marjoram)

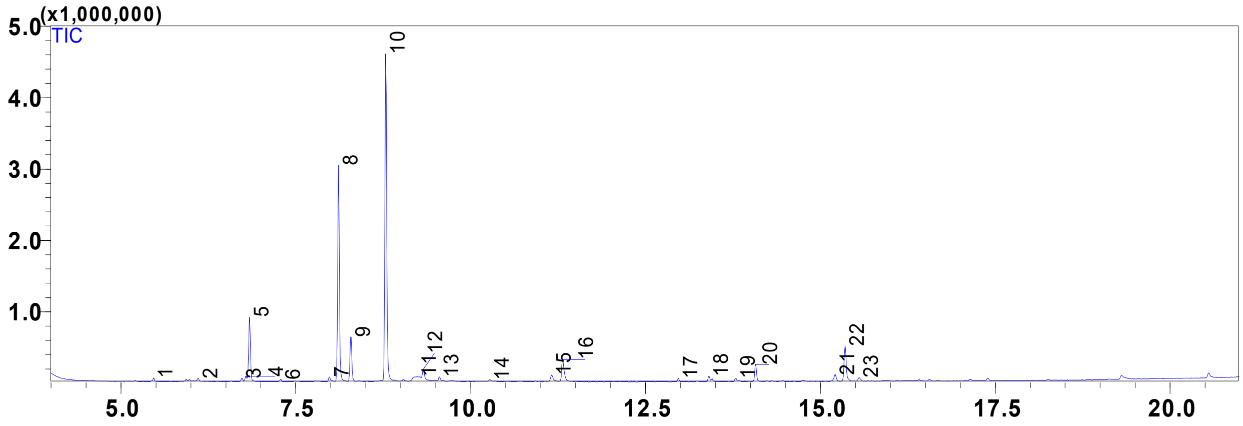

Figure S3. TIC chromatogram of the volatile composition of *O. majorana* EO using GC-MS. Numbers indicate compounds names as in Table S3.

Table S3. Volatile compounds found in the essential oil of *O. majorana.*

No.	Compounds	R.T. (min)	Area (%)
**1**	Camphene	5.466	0.49
**2**	β-Myrcene	6.106	0.47
**3**	*o*-Cymene	6.727	0.39
**4**	D-Limonene	6.787	0.58
**5**	1,8-Cineol	6.839	7.42
**6**	γ-Terpinene	7.284	0.22
**7**	Linalool	7.981	0.80
**8**	3-Thujanone	8.109	23.03
**9**	Thujone	8.287	5.69
**10**	Camphor	8.785	39.58
**11**	Borneol	9.234	1.58
**12**	4-Terpineol	9.321	2.26
**13**	*p*-menth-1-en-8-ol	9.551	0.62
**14**	Pulegone	10.272	0.46
**15**	Carvacrol	11.158	1.27
**16**	Thymol	11.316	4.00
**17**	Caryophyllene	12.970	0.38
**18**	α-Himachalene	13.406	0.78
**19**	Humulene	13.790	0.49
**20**	β-Himachalene	14.073	2.10
**21**	Caryophyllene oxide	15.212	1.23
**22**	Epiglobulol	15.352	5.50
**23**	*cis*-Limonene oxide	15.552	0.66

Camphene

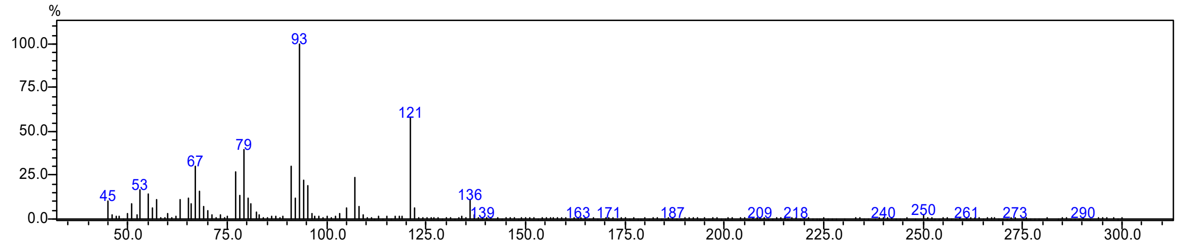

β-Myrcene

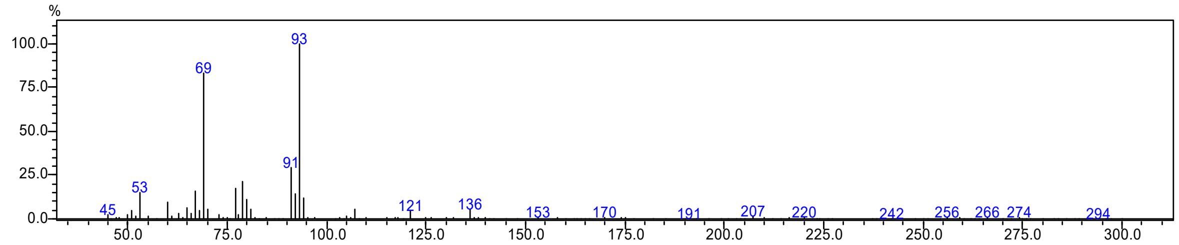

o-Cymene

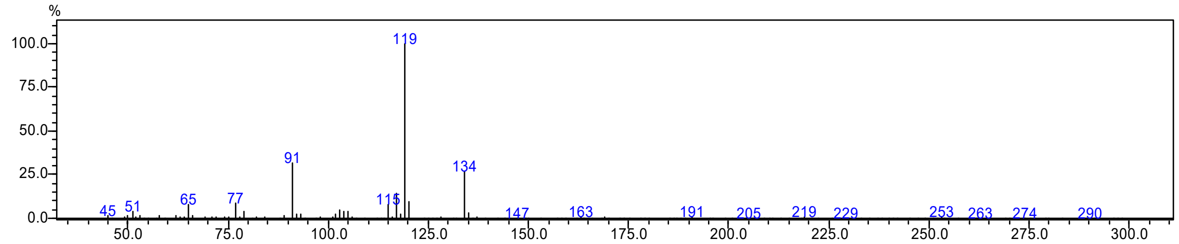

D-Limonene

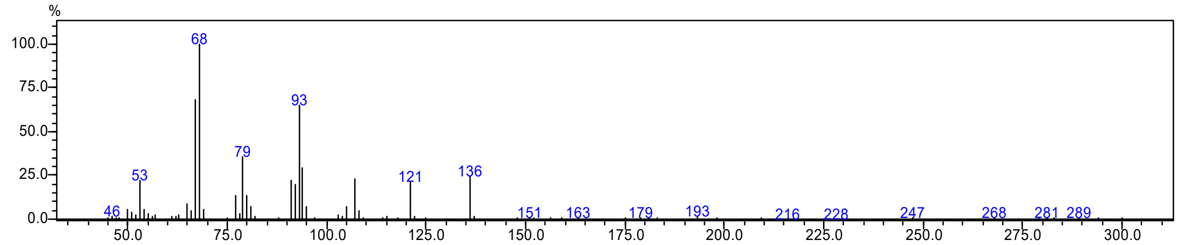

1,8-Cineol

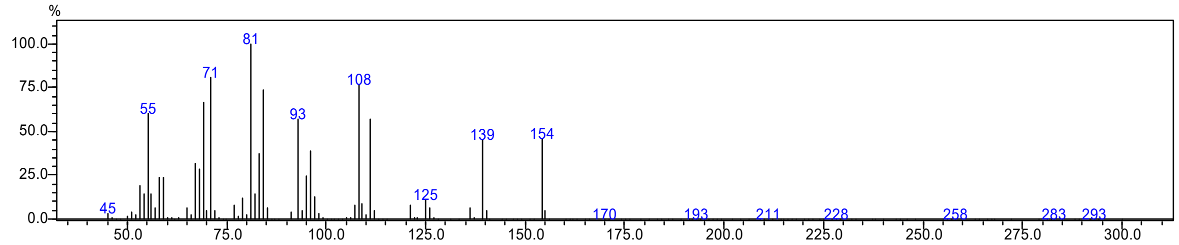

γ-Terpinene

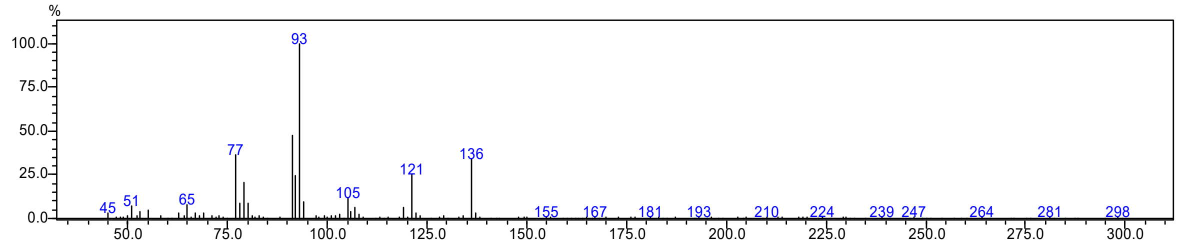

Linalool

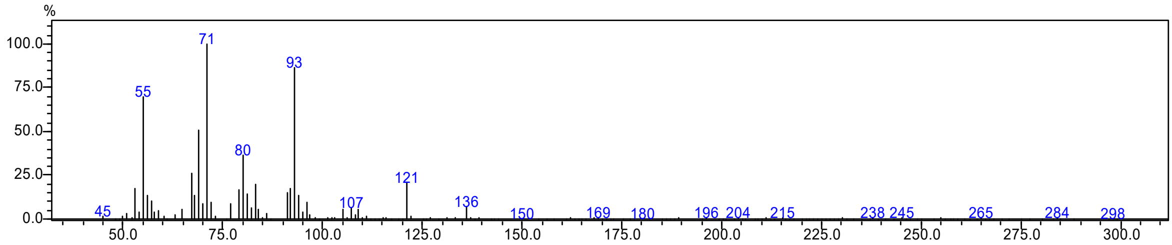

3-Thujanone

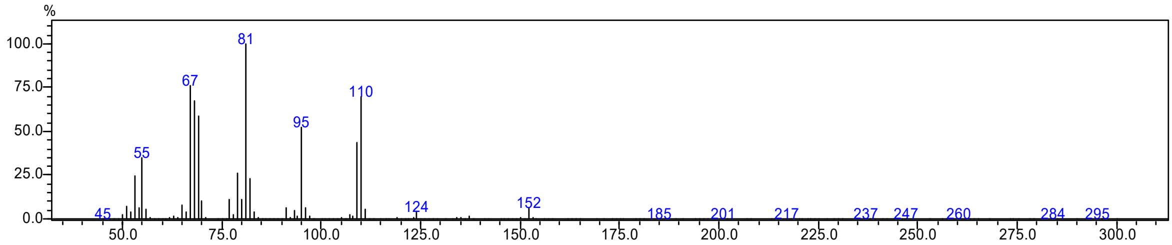

Thujone

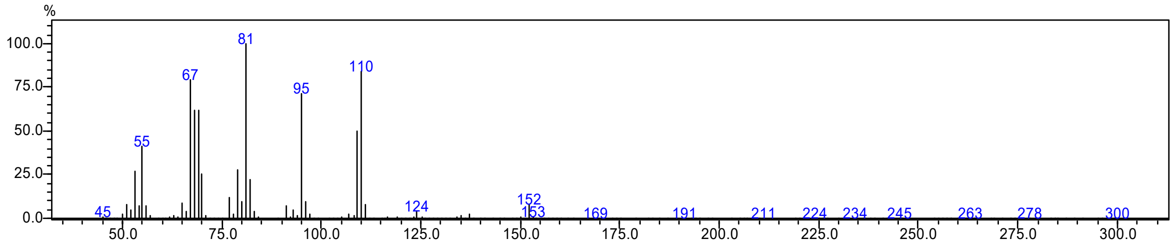

Camphor

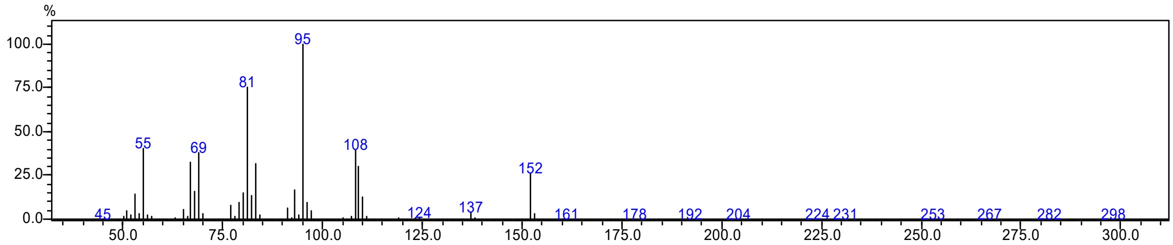

Borneol

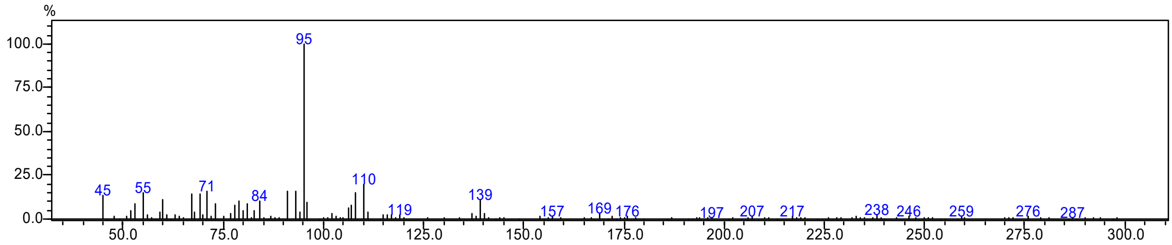

4-Terpineol

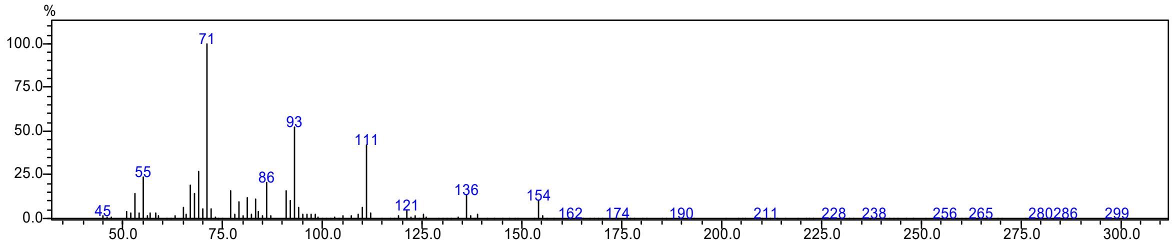

p-menth-1-en-8-ol

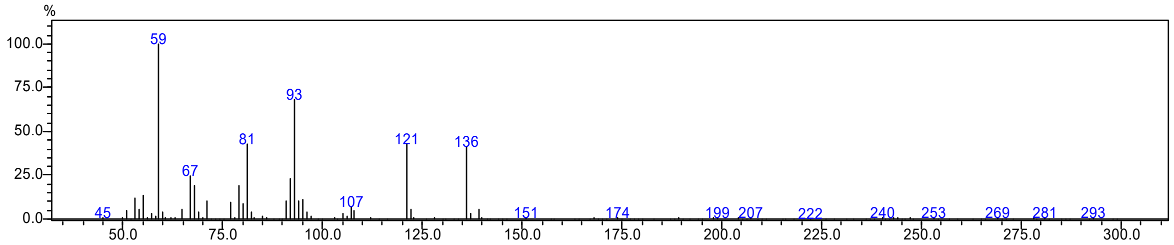

Pulegone

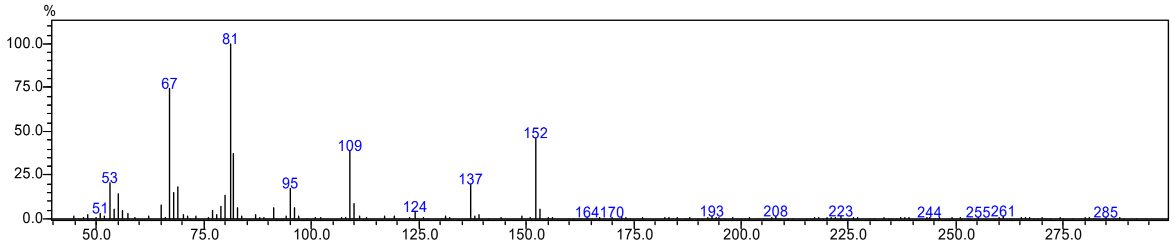

Carvacrol

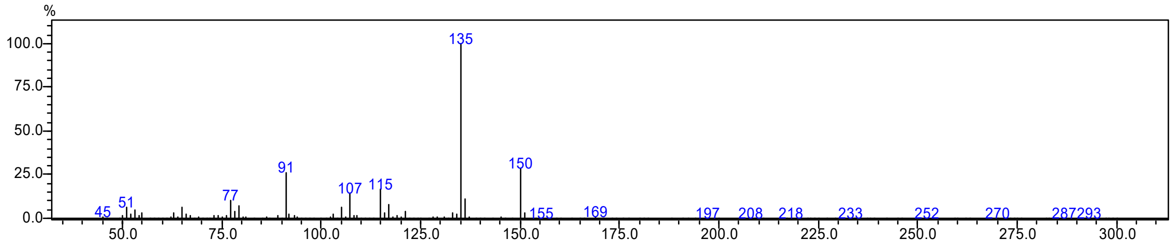

Thymol

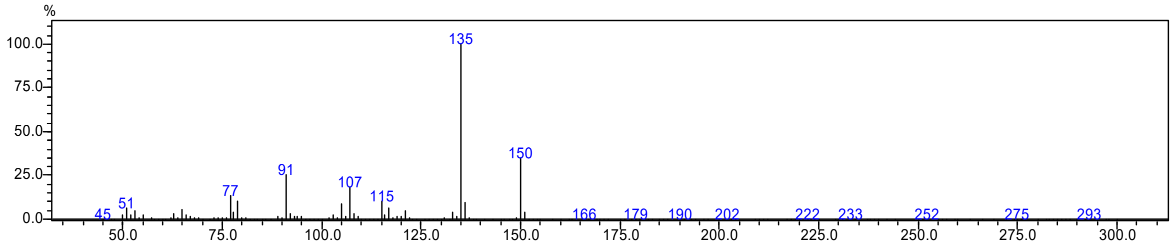

Caryophyllene

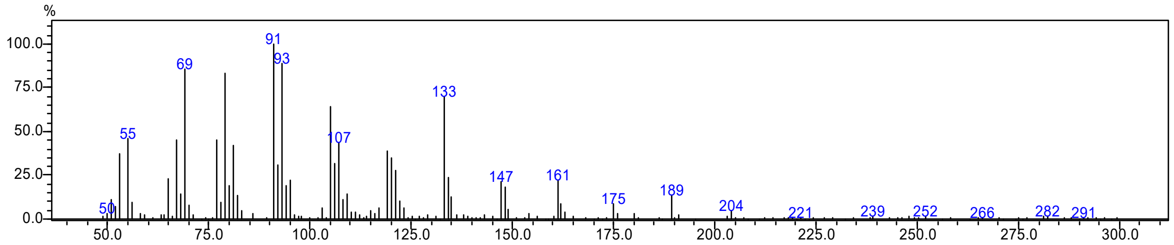

α-Himachalene

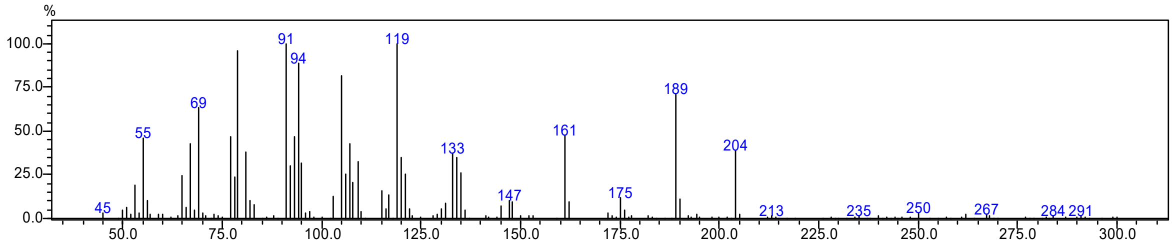

Humulene

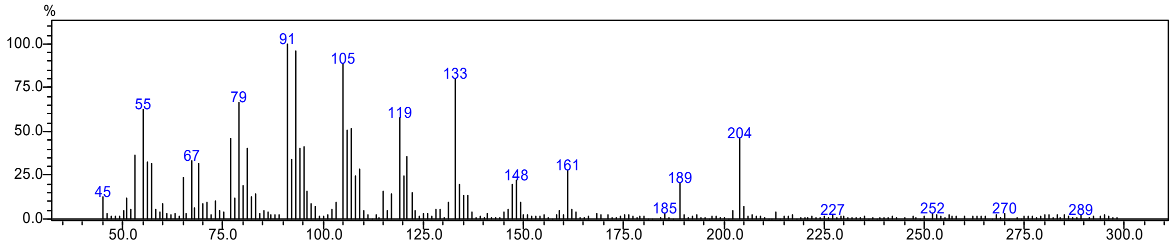

β-Himachalene

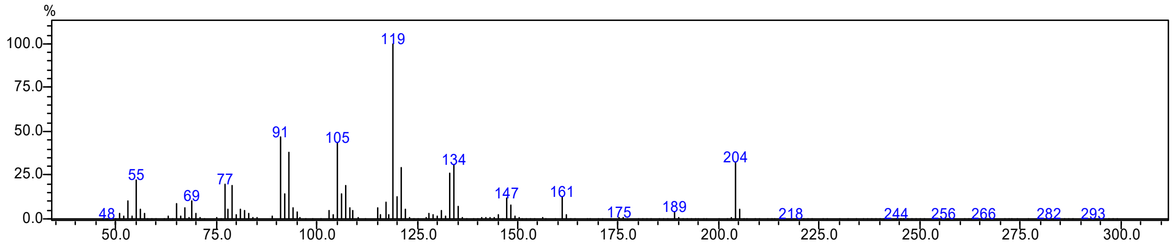

Caryophyllene oxide

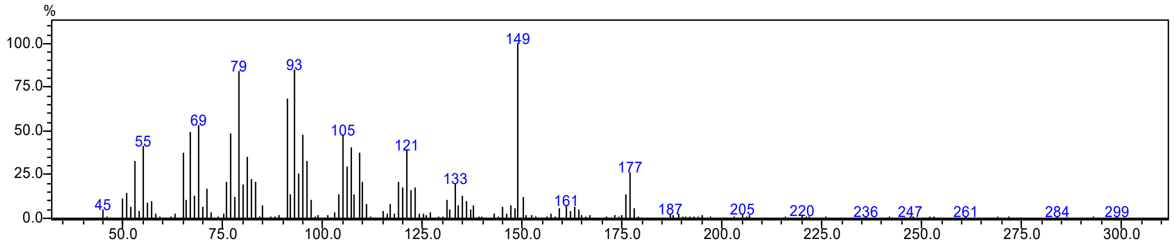

Epiglobulol

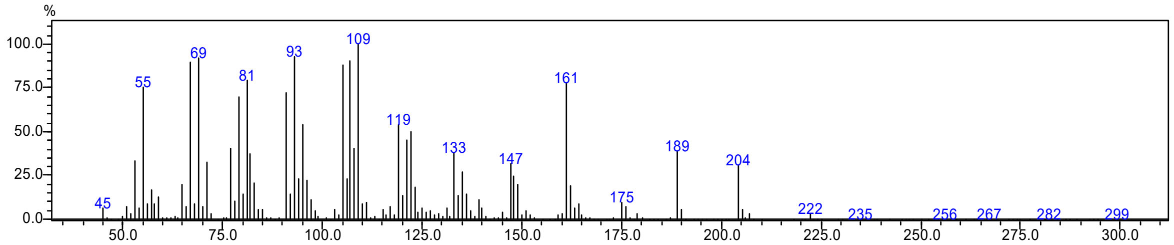

cis-Limonene oxide

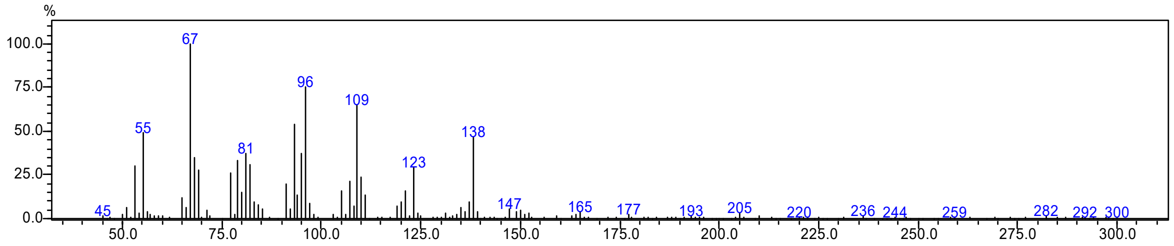

Supplement: Supplementary file 1 [file pharmaceuticals-18-00057-s001.zip › pharmaceuticals-3375568-supplementary.pdf]
